# Integrated image-based deep learning and language models for primary diabetes care

**DOI:** 10.1038/s41591-024-03139-8

**Published:** 2024-07-19

**Authors:** Jiajia Li, Zhouyu Guan, Jing Wang, Carol Y. Cheung, Yingfeng Zheng, Lee-Ling Lim, Cynthia Ciwei Lim, Paisan Ruamviboonsuk, Rajiv Raman, Leonor Corsino, Justin B. Echouffo-Tcheugui, Andrea O. Y. Luk, Li Jia Chen, Xiaodong Sun, Haslina Hamzah, Qiang Wu, Xiangning Wang, Ruhan Liu, Ya Xing Wang, Tingli Chen, Xiao Zhang, Xiaolong Yang, Jun Yin, Jing Wan, Wei Du, Ten Cheer Quek, Jocelyn Hui Lin Goh, Dawei Yang, Xiaoyan Hu, Truong X. Nguyen, Simon K. H. Szeto, Peranut Chotcomwongse, Rachid Malek, Nargiza Normatova, Nilufar Ibragimova, Ramyaa Srinivasan, Pingting Zhong, Wenyong Huang, Chenxin Deng, Lei Ruan, Cuntai Zhang, Chenxi Zhang, Yan Zhou, Chan Wu, Rongping Dai, Sky Wei Chee Koh, Adina Abdullah, Nicholas Ken Yoong Hee, Hong Chang Tan, Zhong Hong Liew, Carolyn Shan-Yeu Tien, Shih Ling Kao, Amanda Yuan Ling Lim, Shao Feng Mok, Lina Sun, Jing Gu, Liang Wu, Tingyao Li, Di Cheng, Zheyuan Wang, Yiming Qin, Ling Dai, Ziyao Meng, Jia Shu, Yuwei Lu, Nan Jiang, Tingting Hu, Shan Huang, Gengyou Huang, Shujie Yu, Dan Liu, Weizhi Ma, Minyi Guo, Xinping Guan, Xiaokang Yang, Covadonga Bascaran, Charles R. Cleland, Yuqian Bao, Elif I. Ekinci, Alicia Jenkins, Juliana C. N. Chan, Yong Mong Bee, Sobha Sivaprasad, Jonathan E. Shaw, Rafael Simó, Pearse A. Keane, Ching-Yu Cheng, Gavin Siew Wei Tan, Weiping Jia, Yih-Chung Tham, Huating Li, Bin Sheng, Tien Yin Wong

**Affiliations:** 1 Shanghai Belt and Road International Joint Laboratory of Intelligent Prevention and Treatment for Metabolic Diseases, Department of Computer Science and Engineering, School of Electronic, Information, and Electrical Engineering, Shanghai Jiao Tong University, Department of Endocrinology and Metabolism, Shanghai Sixth People’s Hospital Affiliated to Shanghai Jiao Tong University School of Medicine, Shanghai Diabetes Institute, Shanghai Clinical Center for Diabetes, Shanghai, China; 2 MOE Key Laboratory of AI, School of Electronic, Information, and Electrical Engineering, Shanghai Jiao Tong University, Shanghai, China; 3 Department of Ophthalmology, Huadong Sanatorium, Wuxi, China; 4 Department of Ophthalmology and Visual Sciences, The Chinese University of Hong Kong, Hong Kong Special Administrative Region, China; 5 State Key Laboratory of Ophthalmology, Zhongshan Ophthalmic Center, Sun Yat-sen University, Guangdong Provincial Key Laboratory of Ophthalmology and Visual Science, Guangdong Provincial Clinical Research Center for Ocular Diseases, Guangzhou, China; 6 Department of Medicine, Faculty of Medicine, University of Malaya, Kuala Lumpur, Malaysia; 7 Department of Renal Medicine, Singapore General Hospital, SingHealth-Duke Academic Medical Centre, Singapore, Singapore; 8 Faculty of Medicine, Department of Ophthalmology, Rajavithi Hospital, College of Medicine, Rangsit University, Bangkok, Thailand; 9 Shri Bhagwan Mahavir Vitreoretinal Services, Medical Research Foundation, Sankara Nethralaya, Chennai, India; 10 Department of Medicine, Division of Endocrinology, Metabolism and Nutrition, and Department of Population Health Sciences, Duke University School of Medicine, Durham, NC USA; 11 Department of Medicine, Division of Endocrinology, Diabetes and Metabolism, Johns Hopkins School of Medicine, Baltimore, MD USA; 12 Department of Medicine and Therapeutics, Prince of Wales Hospital, The Chinese University of Hong Kong, Hong Kong Special Administrative Region, China; 13 Hong Kong Institute of Diabetes and Obesity, The Chinese University of Hong Kong, Hong Kong Special Administrative Region, China; 14 Li Ka Shing Institute of Health Sciences, The Chinese University of Hong Kong, Hong Kong Special Administrative Region, China; 15 Asia Diabetes Foundation, Hong Kong Special Administrative Region, China; 16 Department of Ophthalmology, Shanghai General Hospital, Shanghai Jiao Tong University School of Medicine, Shanghai, China; 17 Singapore Eye Research Institute, Singapore National Eye Centre, Singapore, Singapore; 18 Department of Ophthalmology, Shanghai Sixth People’s Hospital Affiliated to Shanghai Jiao Tong University School of Medicine, Shanghai, China; 19 Beijing Institute of Ophthalmology, Beijing Tongren Hospital, Capital Medical University, Beijing Ophthalmology and Visual Sciences Key Laboratory, Beijing, China; 20 The People’s Hospital of Sixian County, Anhui, China; 21 Department of Endocrinology and Metabolism, Shanghai Eighth People’s Hospital, Shanghai, China; 22 Department of Internal Medicine, Setif University Ferhat Abbas, Setif, Algeria; 23 Ophthalmology Department at Tashkent Advanced Training Institute for Doctors, Tashkent, Uzbekistan; 24 Charity Union of Persons with Disabilities and People with Diabetes UMID, Tashkent, Uzbekistan; 25 Department of Geriatrics, Tongji Hospital, Tongji Medical College, Huazhong University of Science and Technology, Wuhan, China; 26 Department of Ophthalmology, Peking Union Medical College Hospital, Peking Union Medical College, Chinese Academy of Medical Sciences, Beijing, China; 27 National University Polyclinics, National University Health System, Department of Family Medicine, Yong Loo Lin School of Medicine, National University of Singapore, Singapore, Singapore; 28 Department of Primary Care Medicine, Faculty of Medicine, Universiti Malaya, Kuala Lumpur, Malaysia; 29 Department of Medicine, University Malaya Medical Centre, Kuala Lumpur, Malaysia; 30 Department of Endocrinology, Singapore General Hospital, Singapore, Singapore; 31 Division of Endocrinology, University Medicine Cluster, National University Health System, Singapore, Singapore; 32 Department of Medicine, Yong Loo Lin School of Medicine, National University of Singapore, Singapore, Singapore; 33 Department of Internal Medicine, Huadong Sanatorium, Wuxi, China; 34 Institute for AI Industry Research, Tsinghua University, Beijing, China; 35 Department of Automation and the Key Laboratory of System Control and Information Processing, Ministry of Education of China, Shanghai Jiao Tong University, Shanghai, China; 36 International Centre for Eye Health, London School of Hygiene and Tropical Medicine, University of London, London, UK; 37 Department of Endocrinology, Austin Health, Melbourne, Victoria Australia; 38 Department of Medicine, The University of Melbourne (Austin Health), Melbourne, Victoria Australia; 39 Australian Centre for Accelerating Diabetes Innovations, The University of Melbourne, Parkville, Victoria Australia; 40 Baker Heart and Diabetes Institute, Melbourne, Victoria Australia; 41 NHMRC Clinical Trials Centre, University of Sydney, Sydney, New South Wales Australia; 42 NIHR Moorfields Biomedical Research Centre, Moorfields Eye Hospital, London, UK; 43 Centro de Investigación Biomédica en Red de Diabetes y Enfermedades Metabólicas Asociadas, Instituto de Salud Carlos III, Madrid, Spain; 44 Diabetes and Metabolism Research Unit, Vall d’Hebron Research Institut, Autonomous University of Barcelona, Barcelona, Spain; 45 Institute of Ophthalmology, University College London, London, UK; 46 Center for Innovation and Precision Eye Health and Department of Ophthalmology, Yong Loo Lin School of Medicine, National University of Singapore, Singapore, Singapore; 47 Ophthalmology and Visual Science Academic Clinical Program, Duke-NUS Medical School, Singapore, Singapore; 48 School of Clinical Medicine, Tsinghua Medicine, Tsinghua University, Beijing, China; 49 Beijing Tsinghua Changgung Hospital, Beijing, China; 50 Zhongshan Ophthalmic Center, Guangzhou, China

**Keywords:** Diabetes, Diabetes complications

## Abstract

Primary diabetes care and diabetic retinopathy (DR) screening persist as major public health challenges due to a shortage of trained primary care physicians (PCPs), particularly in low-resource settings. Here, to bridge the gaps, we developed an integrated image–language system (DeepDR-LLM), combining a large language model (LLM module) and image-based deep learning (DeepDR-Transformer), to provide individualized diabetes management recommendations to PCPs. In a retrospective evaluation, the LLM module demonstrated comparable performance to PCPs and endocrinology residents when tested in English and outperformed PCPs and had comparable performance to endocrinology residents in Chinese. For identifying referable DR, the average PCP’s accuracy was 81.0% unassisted and 92.3% assisted by DeepDR-Transformer. Furthermore, we performed a single-center real-world prospective study, deploying DeepDR-LLM. We compared diabetes management adherence of patients under the unassisted PCP arm (*n* = 397) with those under the PCP+DeepDR-LLM arm (*n* = 372). Patients with newly diagnosed diabetes in the PCP+DeepDR-LLM arm showed better self-management behaviors throughout follow-up (*P* < 0.05). For patients with referral DR, those in the PCP+DeepDR-LLM arm were more likely to adhere to DR referrals (*P* < 0.01). Additionally, DeepDR-LLM deployment improved the quality and empathy level of management recommendations. Given its multifaceted performance, DeepDR-LLM holds promise as a digital solution for enhancing primary diabetes care and DR screening.

## Abstract (web summary)

Tailored to provide diabetes management recommendations from large training and validation datasets, an artificial intelligence system integrating language and computer vision capabilities is shown to improve self-management of patients in a prospective implementation study.

## Main

It has been estimated that more than 500 million people had diabetes worldwide in 2021, with 80% living in low- and middle-income countries (LMICs)^1,2^. The escalating prevalence imposes a substantial public health challenge, particularly in these low-resource settings^1,3–5^. In LMICs, insufficient healthcare resource and a lack of trained primary care physicians (PCPs) remain principal barriers, resulting in widespread underdiagnosis, poor primary diabetes management and inadequate and/or inappropriate referrals to diabetes specialist care^4,6,7^. This not only impacts on individual health outcomes but also has broader socioeconomic consequences^4,8–10^.

Diabetic retinopathy (DR) is the most common specific complication of diabetes, affecting 30–40% of individuals with diabetes^11–13^, and remains the leading cause of blindness in economically active, working-aged adults^11,14,15^. The presence of DR also signifies a heightened risk of other complications elsewhere (for example, kidney, heart and brain)^16^. Thus, regular DR screening has been universally recommended as a key part of primary diabetes care^17^. However, DR screening is often neglected in low-resource settings in LMICs owing to a scarcity of infrastructure, manpower and sustainable cost-effective DR screening programs.

Several digital technologies have emerged to address gaps in diabetes care and DR screening, including telemedicine^18–20^, artificial intelligence (AI)-assisted glucose monitoring and prediction^21^, retinal image-based deep learning (DL) models^22–24^ and the development of low-cost and portable retinal cameras^25,26^. However, these solutions often focus either on enhancing diabetes management or on providing DR screening but rarely integrate both important aspects for diabetes care. These current solutions also require sufficiently trained PCPs capable of utilizing these digital tools, understanding diabetes care, and referral guidelines for severe DR cases that require specialists interventions, but there are few trained PCPs in low-resource settings^27^.

Recently, large language models (LLMs)^28–31^, achieving natural language understanding and generation, have been developing rapidly and show promise in enhancing healthcare service delivery. LLMs have the potential to optimize patient monitoring, personalization of treatment plans, and patient education, potentially resulting in improved outcomes for patients with diabetes^32–34^ and retinal diseases^35,36^. However, while they perform well in answering some general medical queries^31,37^, current LLMs fall short in providing reliable and detailed management recommendations for major specific diseases^31,38,39^, such as diabetes.

**Fig. 1:**
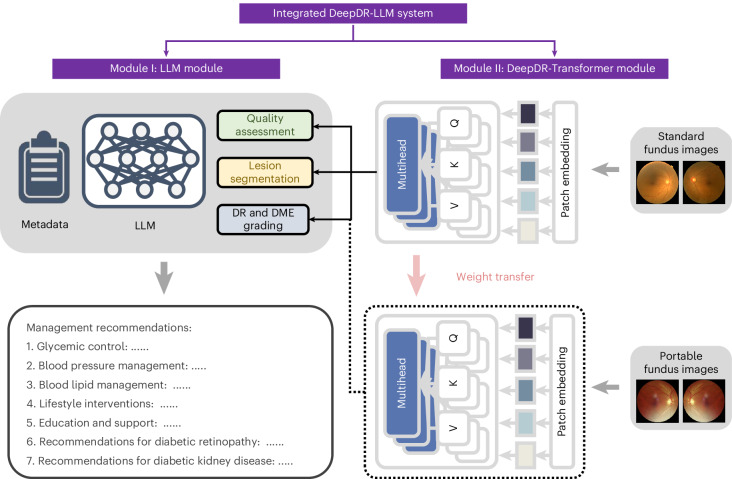
Architecture of the DeepDR-LLM system. The DeepDR-LLM system consists of two modules: (1) module I (LLM module), which provides individualized management recommendations for patients with diabetes; (2) module II (DeepDR-Transformer module), which performs image quality assessment, DR lesion segmentation and DR/DME grading from standard or portable fundus images. There are two modes of integrating module I and module II in the DeepDR-LLM system. In the physician-involved integration mode, the outputs of module II (that is, fundus image gradability; the lesion segmentation of microaneurysm, cotton-wool spot, hard exudate and hemorrhage; DR grade; and DME grade) could assist physicians in generating DR/DME diagnosis results (that is, fundus image gradability, DR grade, DME grade and the presence of lesions). In the automated integration mode, the DR/DME diagnosis results include fundus image gradability, DR grade, DME grade classified by module II, and the presence of lesions segmented out by module II. These DR/DME diagnosis results and other clinical metadata will be fed into module I to generate individualized management recommendations for people with diabetes.

**Fig. 2:**
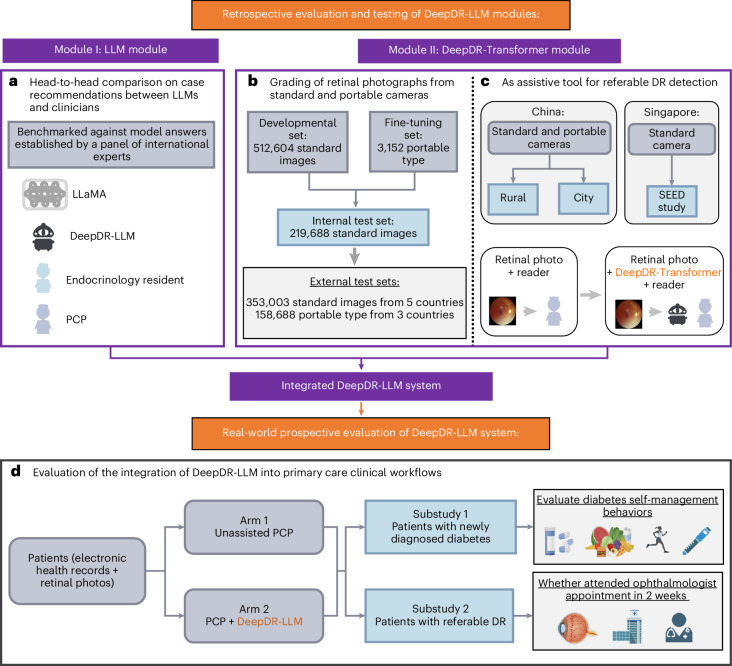
Study design overview for the DeepDR-LLM system evaluation. **a**, Head-to-head comparative assessment of diabetes management recommendations generated by DeepDR-LLM, nontuned LLaMA, PCPs and endocrinology residents, using 100 cases randomly selected from CNDCS. **b**, Efficacy analysis of the DeepDR-Transformer module on multiethnic datasets of standard and portable fundus images. **c**, Utility evaluation of the DeepDR-Transformer module as an assistive tool for PCPs and professional graders in the detection of referable DR. **d**, Study design of a two-arm, real-world, prospective study to evaluate the impact of DeepDR-LLM on patients’ self-management behavior. In the outcome analysis, for substudy I, 253 participants in the unassisted PCP arm and 234 participants in the PCP+DeepDR-LLM arm were included; for substudy II, 154 participants in the unassisted PCP arm and 144 participants in the PCP+DeepDR-LLM arm were included.

To address these interrelated gaps in diabetes care, we developed an innovative image–language system—DeepDR-LLM—which integrates an LLM module with an image-based DL module to offer a comprehensive approach for primary diabetes care and DR screening. Our system is tailored for PCPs, particularly those working in high-volume and low-resource settings. The DeepDR-LLM system comprises two core components: an LLM module and an image-based DL module, referred to as DeepDR-Transformer (Fig. 1). Our evaluation of DeepDR-LLM’s performance relied on four experiments outlined in Fig. 2a–d. First, we developed the LLM module by fine-tuning LLaMA^38^, an open-source LLM that used 371,763 real-world management recommendations from 267,730 participants. We then performed a head-to-head comparative analysis, where we examined the system’s LLM module’s proficiency in providing evidence-based diabetes management recommendations against that of LLaMA, PCPs and in-training specialists (endocrinology residents), with assessments conducted in both English and Chinese languages (Fig. 2a). Second, we trained and tested the performance of DeepDR-Transformer for referable DR detection, using multiethnic, multicountry datasets comprising 1,085,295 standard (table-top) and 161,840 portable (mobile) retinal images (Fig. 2b). Third, we evaluated the impact of DeepDR-Transformer in assisting PCPs and professional graders to identify referable DR (Fig. 2c). Finally, we conducted a two-arm, real-world prospective study to determine the impact of DeepDR-LLM system when integrated into clinical workflow in the primary care setting. Over a 4-week period, we monitored and compared the adherence to diabetes management recommendations between patients under the care of unassisted PCPs and those under the care of PCPs assisted by DeepDR-LLM (Fig. 2d). Collectively, our work offers a digital solution for primary diabetes care combining DR screening and referral, particularly useful in high-volume, low-resource settings in LMICs.

## Results

### Study design and participants

**Extended Data Fig. 1:**
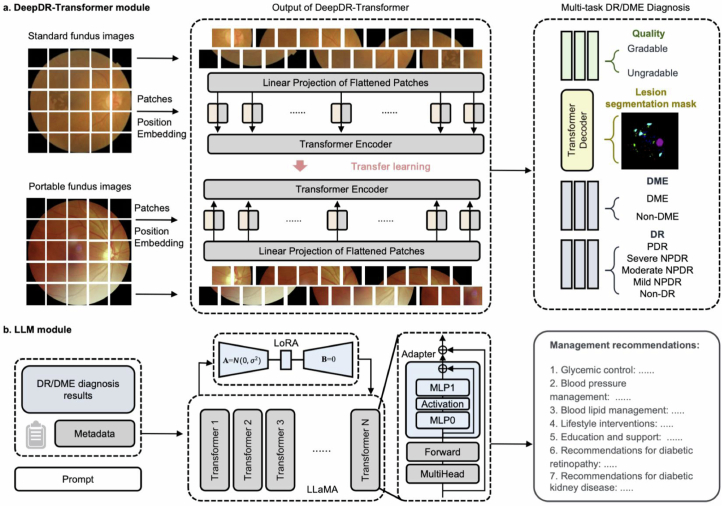
Schematic overview of the DeepDR-LLM system. **a**, Model architecture of the DeepDR-Transformer module. **b**, Model architecture of the LLM module. DME, diabetic macular edema; DR, diabetic retinopathy; NPDR, non-proliferative diabetic retinopathy; PDR, proliferative diabetic retinopathy; LLM, large language model; LoRA, Low-Rank Adaptation; MLP, Multi-Layer Perceptron.

The DeepDR-LLM system consists of two modules: (1) module I (the LLM module), which provides individualized management recommendations for patients with diabetes; (2) module II (the DeepDR-Transformer module), which performs image quality assessment, lesion segmentation and DR grading from standard or portable fundus images for each patient. The outputs of module II (results of real-time DR screening) can also be used as inputs for the LLM module (module I). Extended Data Fig. 1 depicts a schematic overview of the DeepDR-LLM system.

The LLM module was retrospectively evaluated in head-to-head comparisons against the nontuned LLaMA by PCPs and endocrinology residents, in both English and Chinese languages.

The DeepDR-Transformer module was developed and validated in 14 datasets across 5 countries (China, Singapore, India, Thailand and the UK) with standard fundus images, and 7 datasets across 3 countries (China, Algeria and Uzbekistan) with portable fundus images. The characteristics of the datasets are summarized in Supplementary Tables 1 and 2.

### Performance of the LLM module (experiment 2a)

**Extended Data Fig. 2:**
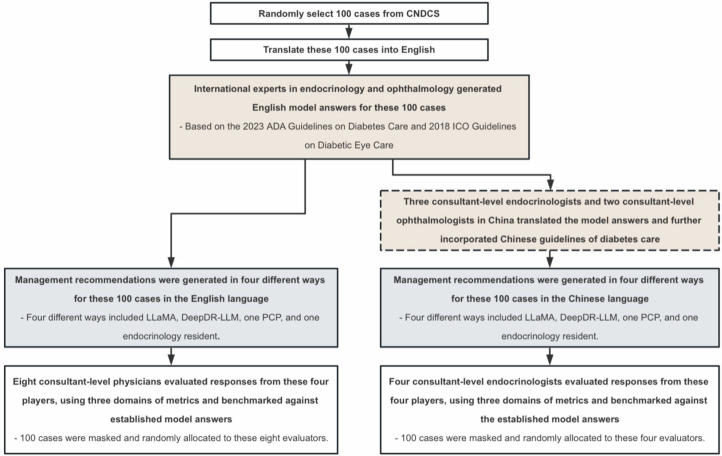
Study design of head-to-head comparison on diabetic management recommendations between large language models (DeepDR-LLM and LLaMA) and clinicians in both English and Chinese languages. CNDCS, China National Diabetic Complications Study; PCP, primary care physician; ADA, American Diabetes Association; ICO, International Council of Ophthalmology.

To evaluate the DeepDR-LLM system’s proficiency in providing diabetes management recommendations in both English and Chinese languages, we compared DeepDR-LLM against LLaMA, PCPs and endocrinology residents on the basis of 100 cases randomly selected from China National Diabetic Complications Study (CNDCS) (Supplementary Table 3 and Extended Data Fig. 2). The recommendations were evaluated on the basis of three axes, namely the extent of inappropriate content, extent of missing content and likelihood of possible harm (Supplementary Table 4).

**Fig. 3:**
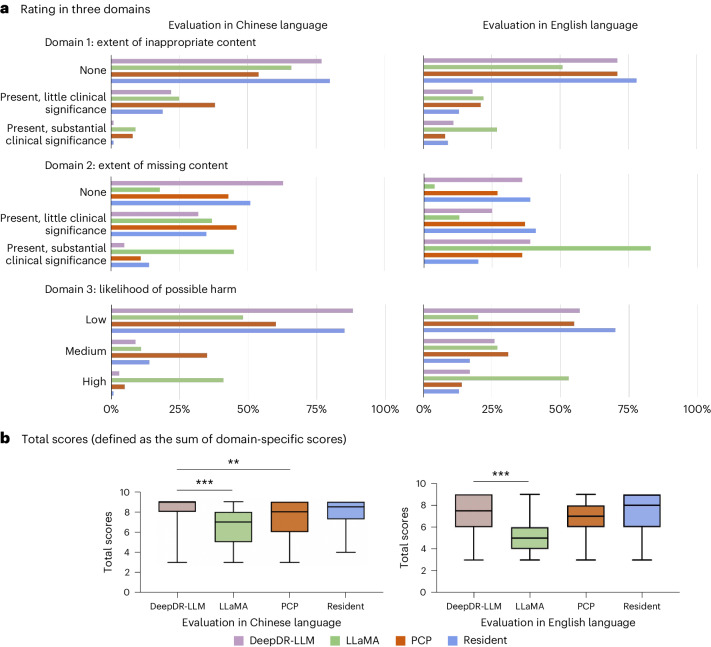
Head-to-head comparison between DeepDR-LLM, nontuned LLaMA, PCP and endocrinology resident in both English and Chinese. **a**, Evaluators were invited to rate management recommendations for patients with diabetes, based on three domains, namely the extent of inappropriate content, the extent of missing content and the likelihood of possible harm, using 100 cases randomly selected from CNDCS. **b**, The total scores of management recommendations generated by LLaMA, DeepDR-LLM, PCPs and endocrinology residents, using 100 cases randomly selected from CNDCS. Box plot (*n* = 100), median and quartiles; whiskers, data range. The comparison was performed using two-sided Friedman tests. Post-hoc pairwise comparisons were performed using two-sided Wilcoxon signed-rank tests. *P* values for multiple comparisons were adjusted using the Bonferroni method. **P = 0.010, ***P < 0.001. Source data

Figure 3a reports evaluations of diabetes management recommendations generated in four different ways (DeepDR-LLM, LLaMA, PCP and resident) summarized into three different domains (inappropriate content, missing content and likelihood of possible harm) in both English and Chinese languages. In English, 71% of DeepDR-LLM recommendations were judged to have no inappropriate content, higher than LLaMA (51%), but comparable to the PCP (71%). In addition, 36% of DeepDR-LLM recommendations were judged not to have missing content (PCP: 27%). Lastly, 57% of DeepDR-LLM recommendations were rated as ‘low likelihood’ for possible harm, comparable to 55% in PCP. In Chinese, 77% of DeepDR-LLM recommendations were judged to have no inappropriate content, higher than LLaMA (66%) and PCP (54%). Additionally, 63% of DeepDR-LLM recommendations were judged not to have missing content, compared to 46% in PCP. Eighty-eight percent of DeepDR-LLM recommendations were rated as ‘low likelihood’ for possible harm, compared to 60% in PCP.

Figure 3b shows the total scores (defined as the sum of domain-specific scores) of the management recommendations generated in four different ways. In English, management recommendations given by DeepDR-LLM were significantly better than those given by LLaMA (*P* < 0.001) and comparable to the PCP and endocrinology resident. In Chinese, management recommendations given by DeepDR-LLM were significantly better than those by LLaMA (*P* < 0.001) and PCP (*P* = 0.010) but comparable to the endocrinology resident.

### Multiethnic validation of DeepDR-Transformer (experiment 2b)

The DeepDR-Transformer module was retrospectively developed and validated in 14 datasets with standard fundus images and 7 datasets with portable fundus images. The characteristics of datasets used in the performance evaluation of DeepDR-Transformer are summarized in Supplementary Tables 1 and 2.

Supplementary Tables 5 and 6 summarize the performances of DeepDR-Transformer in image quality assessment and lesion segmentation. For DR grading, we assessed the performance of the DeepDR-Transformer model in detecting early-to-late stages of DR (multiclass) from standard fundus images and referable DR from portable fundus images (Supplementary Table 7). In standard fundus images, the DeepDR-Transformer model showed excellent performance in identifying referable DR, with areas under the receiver operating characteristic curve (AUCs) ranging from 0.892 to 0.933 across 12 external test sets. In portable fundus images, the model showed AUCs ranging from 0.896 to 0.920 across six external test sets.

### DeepDR-Transformer as an assistive tool (experiment 2c)

**Fig. 4:**
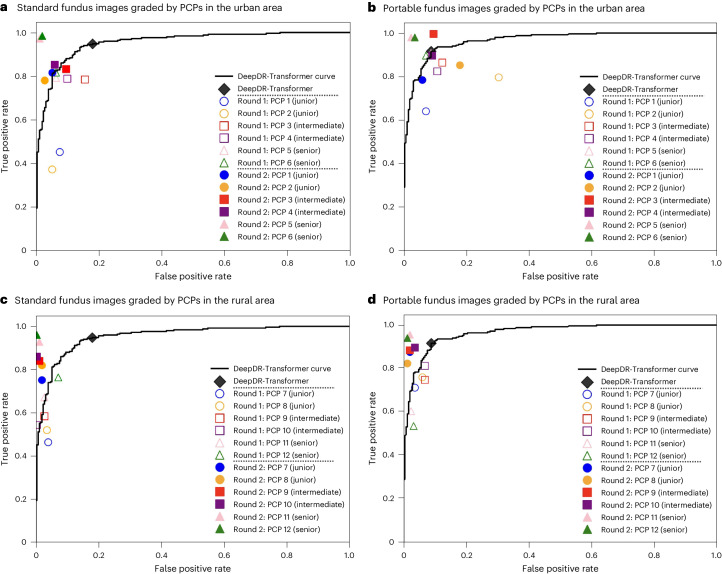
Receiver operating characteristic curves showing performance of DeepDR-Transformer alone versus PCPs (when unassisted and assisted by DeepDR-Transformer) in identifying referable DR. **a**, Standard fundus images (500 eyes: 250 nonreferable eyes and 250 referable eyes) graded by PCPs in the urban area. **b**, Portable fundus images (500 eyes: 250 nonreferable eyes and 250 referable eyes) graded by PCPs in the urban area. **c**, Standard fundus images (500 eyes: 250 nonreferable eyes and 250 referable eyes) graded by PCPs in the rural area. **d**, Portable fundus images (500 eyes: 250 nonreferable eyes and 250 referable eyes) graded by PCPs in the rural area. Source data

To evaluate DeepDR-Transformer as an assistive tool for PCPs and professional nonphysician graders (these graders are now used in many DR screening programs, such as the UK, Singapore and Vietnam, in place of PCPs^40–43^) in identifying referable DR, we assessed both the accuracy and time efficiency of the grading processes with and without the assistance of the DeepDR-Transformer module (Fig. 4, Extended Data Tables 1–3 and Supplementary Fig. 1). Based on standard fundus images graded by PCPs in the urban area (Fig. 4a and Extended Data Table 1), we observed a sensitivity range of 37.2–81.6% for unassisted PCPs, which subsequently increased to 78.0–98.4% with DeepDR-Transformer assistance. Similarly, specificity improved from the original range of 84.4–94.8% (unassisted) to 90.4–98.8% when assisted with DeepDR-Transformer. Moreover, with the assistance of DeepDR-Transformer, the median time taken for assessment was reduced from 14.66 s (interquartile range (IQR) 14.09–15.57) per eye to 11.31 s (IQR 10.82–11.84) (*P* < 0.001), indicating a significant enhancement in both the accuracy and efficiency of DR grading.

Because table-top-mounted retinal cameras require more space and are typically more expensive, we conducted another experiment using portable retinal cameras. For portable fundus images graded by PCPs in the urban area (Fig. 4b and Extended Data Table 3), the sensitivity ranged from 64.0% to 90.8% for unassisted PCPs, which subsequently increased to 78.4% to 99.6% with DeepDR-Transformer assistance. Specificity improved from the original range of 69.6–92.8% (unassisted) to 82.0–97.6% with DeepDR-Transformer assistance. Furthermore, the median time taken for assessment was reduced from 7.39 s (IQR 6.69–8.42) per eye to 6.13 s (IQR 5.82–6.73) with DeepDR-Transformer’s assistance (*P* < 0.001). Similar trends were observed in standard and portable fundus images graded by PCPs in the rural area (Fig. 4c,d and Extended Data Tables 1 and 3).

### Prospective real-world study of DeepDR-LLM (experiment 2d)

**Extended Data Fig. 3:**
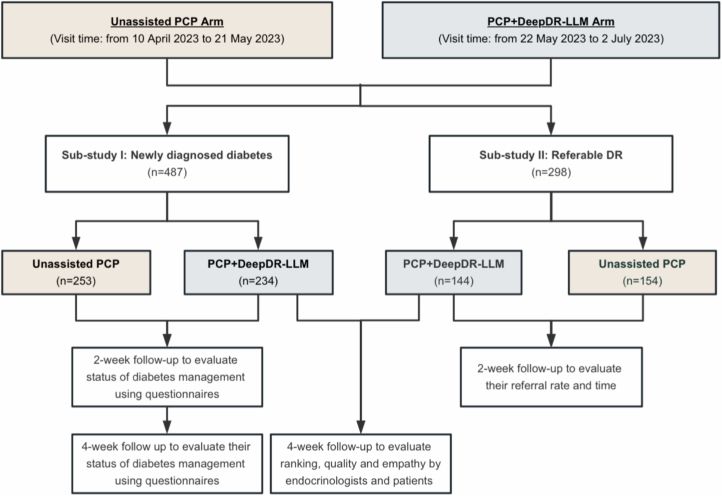
Study design of the real-world, two-arm, prospective study. For patients in the unassisted PCP arm, ten patients were diagnosed with both newly diagnosed diabetes and referable DR. For patients in the PCP+DeepDR-LLM arm, six patients were diagnosed with both newly diagnosed diabetes and referable DR. PCP, primary care physician; DR, diabetic retinopathy.

To evaluate the impact of implementing the integrated DeepDR-LLM system (combining both the LLM and DeepDR-Transformer modules), on diabetes self-management behaviors, we carried out a proof-of-concept, two-arm, prospective study in a real-world setting. Extended Data Fig. 3 shows the study design of this real-world prospective study (showing numbers of participants included in the outcome analysis). Participants were allocated to two groups: one receiving management recommendations from PCPs without the assistance of DeepDR-LLM (referred to as the unassisted PCP arm) and the other receiving augmented input where PCPs’ recommendations were enhanced with insights from DeepDR-LLM (referred to as the PCP+DeepDR-LLM arm). Comparisons of baseline characteristics of included participants in two substudies between the two arms are presented in Extended Data Table 4.

For patients diagnosed with newly diagnosed diabetes at baseline, they were followed up after 2 weeks and 4 weeks to evaluate their self-management practices. Patients in the PCP+DeepDR-LLM arm showed better self-management of diabetes in several aspects at the 2-week follow-up, including decreased consumption of refined grains and alcohol, increased consumption of whole grains and fresh vegetables, increased physical activities and adherence to drug therapy (all *P* < 0.05, after adjusting for age, sex and baseline HbA1c level; Extended Data Table 5). At the 4-week follow-up, participants in the PCP+DeepDR-LLM arm maintained better self-management of diabetes and exhibited behaviors of increased consumption of fresh fruits, decreased consumption of starchy vegetables, more frequent blood glucose monitoring and better adherence to antidiabetic medication, compared to those in the unassisted PCP arm (all *P* < 0.05, after adjusting for age, sex and baseline HbA1c level).

For patients diagnosed with referable DR at baseline visit, the 2-week follow-up revealed a significantly positive trend. Those patients in the PCP+DeepDR-LLM arm were more likely to follow through with their referral and consult an ophthalmologist within 2 weeks (77.78% versus 58.44%; *P* = 0.001, as indicated in Extended Data Table 5). Furthermore, patients in the PCP+DeepDR-LLM arm scheduled their post-referral ophthalmologist appointments significantly sooner than those in the unassisted PCP arm (4 (IQR 3–5) days versus 7 (IQR 6–8) days; *P* < 0.001). These findings underscore the positive influence of the integrated DeepDR-LLM system in fostering more proactive self-management actions.

**Fig. 5:**
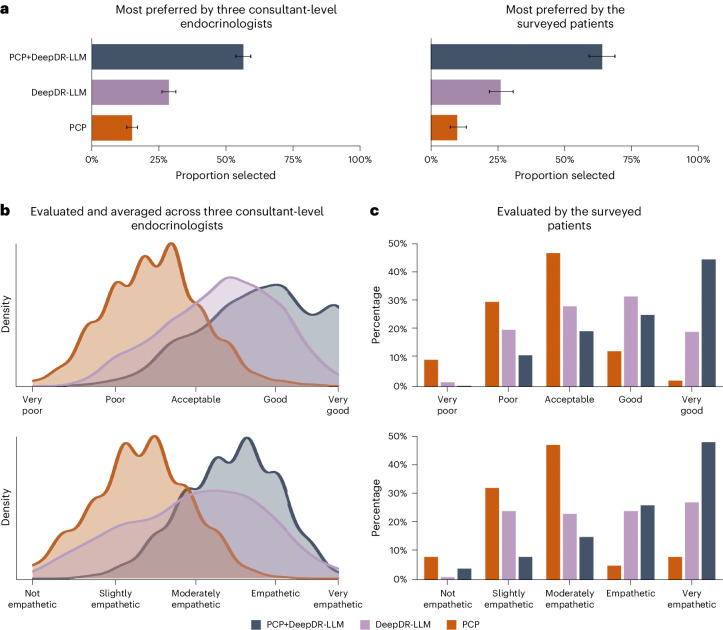
Quality and empathy ratings of the diabetes management recommendations by three consultant-level endocrinologists and 372 surveyed patients in the PCP+DeepDR-LLM arm. **a**, Proportions of PCP, DeepDR-LLM and PCP+DeepDR-LLM’s recommendations being selected as the first-choice preference by consultant-level endocrinologists and patients (number of cases 372). Each of the three consultant-level endocrinologists was invited to evaluate all the 372 cases. The error bars show the Clopper–Pearson 95% CIs. **b**, Kernel density plots showing the quality and empathy ratings of PCP, DeepDR-LLM and PCP+DeepDR-LLM’s recommendations, as evaluated by three consultant-level endocrinologists (number of cases 372). Each of the three consultant-level endocrinologists was invited to evaluate all the 372 cases. **c**, Bar plots showing the quality and empathy ratings of PCP, DeepDR-LLM and PCP+DeepDR-LLM’s recommendations, as evaluated by the 372 surveyed patients (number of cases 372). Source data

**Extended Data Fig. 4:**
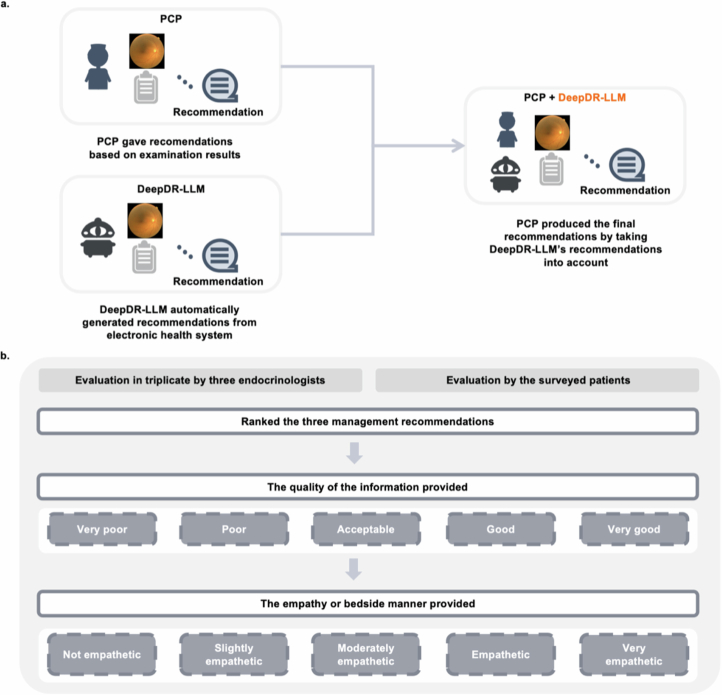
Study design of the post-deployment evaluation of management recommendations’ quality and level of empathy. **a**, In the PCP + DeepDR-LLM arm, the DeepDR-LLM system was integrated into the clinical workflow. Initially, PCPs and DeepDR-LLM gave management recommendations independently. The recommendations given by DeepDR-LLM was automatically generated from electronic health systems, by extracting and analyzing the fundus images, medical history, physical examinations, and laboratory tests. Subsequently, PCPs edited their recommendations in text form by taking DeepDR-LLM’s recommendations into account. **b**, For participants in the PCP + DeepDR-LLM arm, they filled out a questionnaire investigating their opinions on three recommendations at the 4-week follow-up. Evaluators, including endocrinologists and surveyed participants, ranked these three recommendations and judged both ‘the quality of information provided’ (very poor, poor, acceptable, good, or very good) and ‘the empathy or bedside manner provided’ (not empathetic, slightly empathetic, moderately empathetic, empathetic, and very empathetic).

In addition, we carried out a post-deployment evaluation to assess the quality and level of empathy provided by the DeepDR-LLM system alone, PCP alone and PCP+DeepDR-LLM (Extended Data Fig. 4). This evaluation involved three consultant-level endocrinologists and 372 patients. Across these 372 cases evaluated by the three endocrinologists, of the three versions of management recommendations, PCP+DeepDR-LLM’s recommendations were most preferred (56.36%; Fig. 5a) by the endocrinologists. In total, 68.37% of PCP+DeepDR-LLM’s recommendations were rated as either ‘good’ or ‘very good’ quality, and 71.06% recommendations were deemed ‘empathetic’ or ‘very empathetic’ (Fig. 5b). From the patients’ perspective, the majority (238/372, 63.98%) also favored PCP+DeepDR-LLM’s recommendations over the other two versions (Fig. 5a). Similarly, 69.35% of PCP+DeepDR-LLM’s recommendations were rated by the surveyed patients as either ‘good’ or ‘very good’ quality, and 73.92% recommendations were deemed ‘empathetic’ or ‘very empathetic’ (Fig. 5c).

Finally, to capture the PCPs’ perceptions and satisfactions towards the DeepDR-LLM system after using its insights, the 12 PCPs who participated in the PCP+DeepDR-LLM arm of the real-world prospective study were also asked to complete a user satisfaction questionnaire. This questionnaire was completed within 2 weeks after the study closure (Extended Data Table 6). Across the 12 PCPs, the DeepDR-LLM system obtained an average score of 4.42 for being understandable (out of 5.00), 4.33 for time-saving, 4.17 for effectiveness and 4.17 for being safe in clinical practice. It also obtained an overall satisfaction score of 4.50.

## Discussion

Primary diabetes care that is accessible, timely and appropriate persists as a major public health challenge due to insufficient healthcare infrastructure and a lack of trained PCPs, particularly in low-resource settings in many LMICs^4^. Adding to this complexity in primary diabetes care is the need to manage diabetes complications, such as DR, the most specific complication, with its presence often signaling other complications in major organ systems (for example, kidney, heart and brain)^11,12^. While DR screening has been widely recommended by international guidelines, such programs are lacking in low-resource settings due to the scarcity of infrastructure and a lack of trained PCPs who can administer and manage such programs. To address these gaps, we developed an integrated image–language system (DeepDR-LLM) combining an LLM module and a DL module (DeepDR-Transformer), with an aim to provide tailored personalized diabetes management recommendations and real-time fully automated DR screening and referral recommendations to aid the PCPs working in primary diabetes care.

Key features and findings of our system should be emphasized. First, our LLM module was fine-tuned on an open-source LLM (using more than 300,000 real-world management recommendations from more than 250,000 participants), focusing on providing individualized and reliable management recommendations for the PCPs to manage common scenarios in diabetes. In our head-to-head analysis (experiment 2a), we showed that our LLM module performed better than nontuned ‘generic’ LLMs (that is, LLaMA) and PCPs, and with comparable performance to endocrinology residents. Furthermore, our two-arm, real-world prospective study in a primary diabetes care context demonstrated that the integration of DeepDR-LLM with PCP consultations enhanced self-management behaviors in newly diagnosed patients with diabetes and increased adherence to DR referrals for those with identified referable DR.

For the LLM module, in the head-to-head comparison (experiment 2a), we demonstrated that the LLM module of the DeepDR-LLM system could mostly generate reliable management recommendations for patients with diabetes in the retrospective evaluations in both English and Chinese. Previous studies have shown the promising potential of ‘generic’ LLMs in generating answers to real-world consumer queries for medical information, which are usually general and somewhat superficial^31,37^. However, previous LLMs did not provide specific and detailed management recommendations for patients with common diseases^31,38,39^, such as diabetes. Another limitation of previous head-to-head evaluations between LLMs and clinicians was the lack of model answers serving as benchmarks to compare the performance of different players^31^. In our study, we enlisted an international panel of experts in endocrinology and ophthalmology (names listed in Methods) to formulate the model answers for each case, using established clinical guidelines (that is, 2023 American Diabetes Association Guidelines on Diabetes Care^44^ and 2018 International Council of Ophthalmology Guidelines on Diabetic Eye Care^17^). Encouragingly, the LLM module showed performance comparable to endocrinology resident in Chinese and PCPs in English, in all three evaluated axes. These results demonstrated the potential of the DeepDR-LLM system to provide reliable management recommendations for PCPs to manage patients with diabetes.

With respect to the image-based DL component for DR screening, the DeepDR-Transformer module provided robust performance of DR grading in diverse multiethnic cohorts of patients with diabetes (experiment 2b). Importantly, we demonstrated this performance in both standard (desktop) and portable (mobile) fundus images. Existing DL systems for DR screening primarily focused on standard retinal images taken with more expensive desktop fundus cameras^22–24^. In this study, we showed that DeepDR-Transformer could also achieve optimal performance in lower-resolution portable fundus images, with AUCs ranging from 0.896 to 0.920 for detecting referable DR across six external test datasets from China, Algeria and Uzbekistan. The robustness and generalizability of the DeepDR-Transformer module for identifying referable DR from portable fundus images could potentially empower point-of-care DR screening by PCPs in lower-resourced settings, where future DR screening models will probably involve such smaller, cheaper fundus cameras rather than standard retinal cameras^45^.

Finally, to further demonstrate the impact of DeepDR-LLM on patients’ self-management behavior for diabetes care (experiment 2d), we conducted a two-arm, real-world prospective study in a primary care setting. In the unassisted PCP arm, PCPs gave the management recommendations without the help of DeepDR-LLM. We found that these recommendations given by PCPs were generally rule-based with ‘one-size-fits-all’ treatment targets and lifestyle interventions, with little personalization (examples shown in Supplementary Table 8). These findings are probably explained by routine generic answers provided by PCPs, in part due to the lack of in-depth diabetes-specific training of PCPs, a problem even in high-resource settings^4^. On the other hand, in the PCP+DeepDR-LLM arm, using electronic health records and fundus images, our integrated DeepDR-LLM system could generate good quality and empathetic recommendations. These suggestions were then used by PCPs to formulate management plans for each patient. Evaluations by consultant-level endocrinologists and patients indicated that the integration of DeepDR-LLM could significantly enhance the quality and perceived empathy of the PCPs’ recommendations.

Current digital and AI solutions cannot realize their full potential unless seamlessly integrated into existing clinical workflows^46^. We showed that the integration of the DeepDR-LLM system into primary diabetes care could improve patient outcomes in two aspects. First, for patients with newly diagnosed diabetes, the DeepDR-LLM system could promote better self-management behaviors, including dietary modifications (for example, increased consumption of whole grains and decreased consumption of starchy vegetables), increased physical activities and adherence to antidiabetic medication. Concurrently, for those patients diagnosed with referable DR, receiving recommendations from PCPs that were augmented with DeepDR-LLM’s recommendation could improve the compliance rate of attending the ophthalmologists within 2 weeks, as well as shorten the referral interval. These results highlight the beneficial impact of the integrated DeepDR-LLM system in promoting patient engagement and encouraging more proactive health management behaviors.

**Fig. 6:**
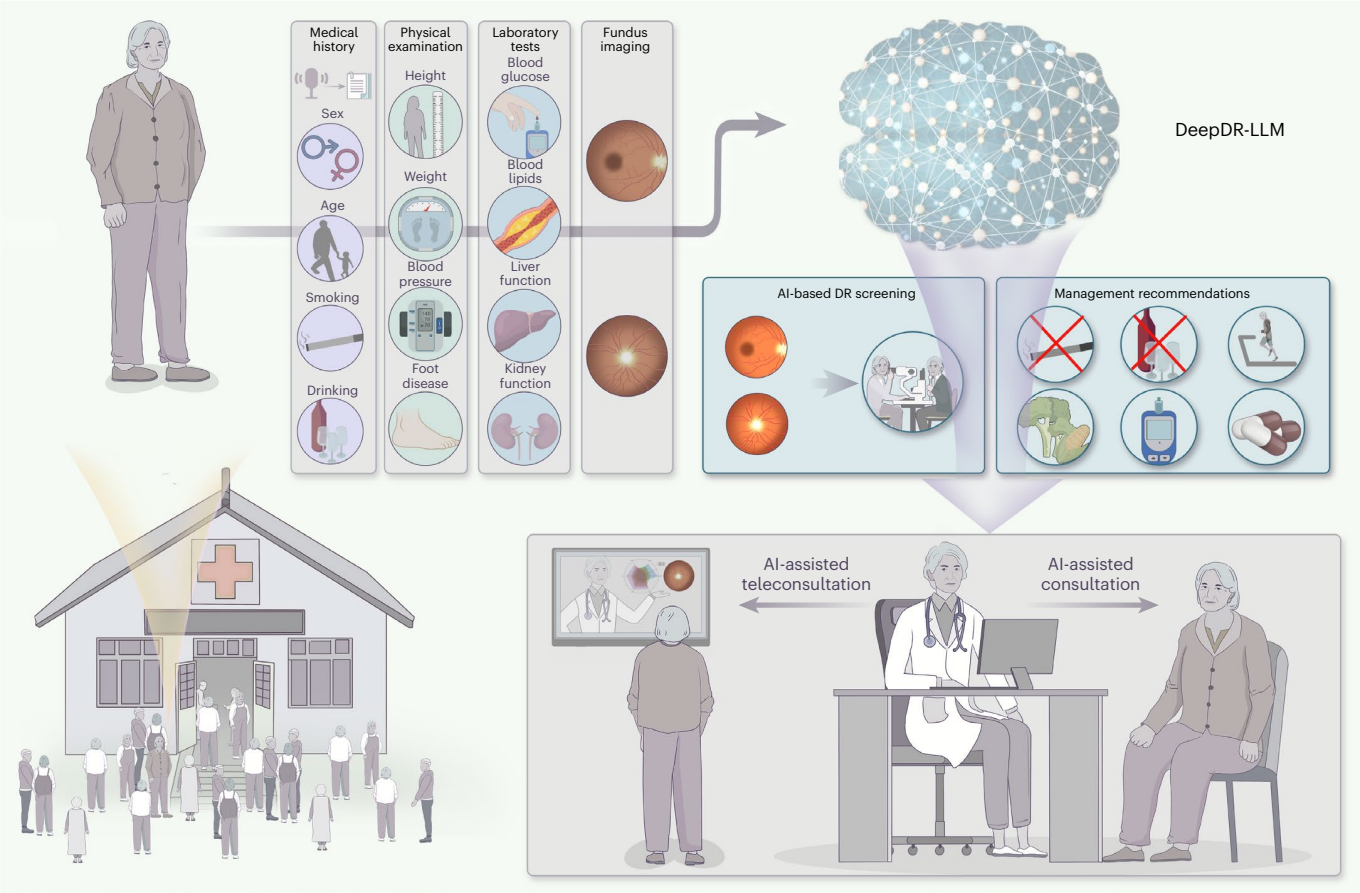
Envisioning the future of primary diabetes care with the clinical integration of the DeepDR-LLM system. First, patients with diabetes undergo comprehensive evaluations that include medical history taking that can be augmented by automated voice-to-text technology, physical examinations, laboratory assessments and fundus imaging. Following this, the DeepDR-LLM system processes the accumulated clinical data to concurrently deliver DR screening results and tailored management recommendations for PCPs. Subsequently, augmented with these AI-derived insights, PCPs then offer treatment guidance and health education to patients, either in person or through teleconsultation services.

For the implementation of digital solutions, feedback from end-users (in this case, PCPs) is critical. In our real-world prospective evaluation of the integrated DeepDR-LLM system, post deployment, most PCPs deemed the system simple and understandable, effective and safe. PCPs who participated in our survey also indicated they would like to use the DeepDR-LLM system in their future practice. Thus, our DeepDR-LLM system holds great potential for primary diabetes care to empower AI-assisted face-to-face consultation or teleconsultation (Fig. 6). Nevertheless, for clinical adoption, other workflow challenges need to be addressed, including addressing data quality issues, ethical, privacy and legal considerations, and integration with existing healthcare information technology infrastructure^47^. Thus, future research directions for DeepDR-LLM should focus on developing more transparent and unbiased datasets applicable to more diverse populations, thereby mitigating data quality issues and the risk of bias and discrimination; exploring ethical and legal frameworks for safe and responsible primary care setting implementation; integration with other technologies (for example, wearables) to further optimize patient engagement; and evaluating the long-term cost-effectiveness and patient outcomes as well as identifying areas for further improvement and refinement^39,48^.

Our study had limitations. First, since our integrated system was trained and fine-tuned exclusively on Chinese populations, additional training or fine-tuning on more diverse clinical and demographic cohorts may further improve the diagnostic accuracy and clinical utility of this system. However, we tested the generalizability of the DeepDR-Transformer module in diverse multiethnic multicountry datasets that showed consistently robust performance across different datasets. Second, the LLM module of the DeepDR-LLM system was evaluated in English and Chinese. Future studies should extend this evaluation to other languages to better assess its broader applicability. Additionally, we did not compare the performance between the LLM module and other open-source LLMs due to concerns about privacy leakage. Third, in the evaluation of the DeepDR-Transformer module of the DeepDR-LLM system as an assistive tool in identifying referable DR, a 1-week washout period between unassisted and DeepDR-Transformer assistant decisions may not be sufficient to fully eliminate the recall bias. Fourth, our real-world prospective evaluation of the DeepDR-LLM system was not designed as a randomized controlled trial, and it primarily focused on self-management behaviors as the key clinical outcomes of interest with a relatively short follow-up period and not sufficiently on objective clinical outcomes (for example, documented progression of DR). As such, the findings of our study could potentially be influenced by sampling bias and self-reporting inaccuracies. Additionally, PCPs were the same in the two arms, which could lead to biases in the intervention due to priori approaches and expectations. Despite these limitations, our study serves as a foundational proof-of-concept that can inform the design of future, prospective or community-based studies or randomized controlled trials. We believe that it is essential to evaluate the longer-term effectiveness of this intervention via future (preferably blinded) randomized studies with a more extended observation period and multiple clinical outcomes (including objective measurements, duration of the consultation interactions, PCPs’ attitude toward the proposed system and subsequent patient outcomes).

In conclusion, we developed an integrated image–language system synergistically combining an LLM module and an image-based DL module (DeepDR-Transformer). We demonstrated that our DeepDR-LLM system could provide personalized high-quality and empathetic management recommendations for patients with diabetes based on their retinal images and routine clinical data. This integrated digital solution could provide complementary functionality to enhance individualized diabetes management and may be useful in low-resource but high-volume settings. Given its multifaceted performance and potential impact, our proposed system holds promise as a digital solution for primary diabetes care management, particularly relevant to 80% of the world’s diabetes population living in underserved, resource-limited settings.

## Methods

### Ethical approval

The study was approved by the Ethics Committee of Shanghai Sixth People’s Hospital (2019-087, approved 29 August 2019; 2023-KY-023(K), approved 7 March 2023; 2023-KY-123(K), approved 5 September 2023) and Huadong Sanatorium (2023-08, approved 2 April 2023). Only deidentified retrospective data were used for the development of the LLM module. For the development and validation of the DeepDR-Transformer module, informed consent was obtained from all participants. For the real-world prospective study, informed consent was obtained from all participants. This study was conducted in accordance with the Declaration of Helsinki.

### Data acquisition and diagnosis criteria

Fourteen independent cross-sectional datasets with standard fundus images and seven independent cross-sectional datasets with portable fundus images from people with diabetes were included in this study. For datasets with standard fundus images, two datasets were used to develop and internally validate the DeepDR-Transformer module: the Shanghai Integration Model (SIM) cohort^24,49^ and the Shanghai Diabetes Prevention Program (SDPP) cohort. In addition, 12 multiethnic datasets were enrolled for external validation: the Nicheng Diabetes Screening Project (NDSP) cohort, the Diabetic Retinopathy Progression Study (DRPS) cohort, the Wuhan Tongji Health Management (WTHM) cohort, the Peking Union Diabetes Management (PUDM) cohort, the CNDCS cohort^50^, the Guangzhou Diabetic Eye Study (GDES) cohort, the Chinese University of Hong Kong-Sight-Threatening Diabetic Retinopathy (CUHK-STDR) cohort^51^, the Singapore Epidemiology of Eye Diseases study (SEED) cohort^22,52^, the Singapore National Diabetic Retinopathy Screening Program (SiDRP) cohort^22^, the Sankara Nethralaya-Diabetic Retinopathy Epidemiology and Molecular Genetics Study (SN-DREAMS) cohort^53^, the Thai National Diabetic Retinopathy Screening Program (TNDRSP) cohort^54^ and United Kingdom Biobank (UKB) cohort. Use of data from the UK Biobank was approved with the UK Biobank Resource under application number 104443.

Portable fundus images from the NDSP cohort were utilized to fine-tune the DeepDR-Transformer module. Another six datasets were included for external validation: the Chinese Portable Screening Study for Diabetic Retinopathy-East (CPSSDRE) cohort, the Chinese Portable Screening Study for Diabetic Retinopathy-Middle (CPSSDRM) cohort, the Chinese Portable Screening Study for Diabetic Retinopathy-West (CPSSDRW) cohort, the Chinese Portable Screening Study for Diabetic Retinopathy-Northeast (CPSSDRN) cohort, the Algerian Diabetic Retinopathy Study (ADRS) cohort and the Uzbek Diabetic Retinopathy Study (UDRS) cohort. The CPSSDRE, CPSSDRM, CPSSDRW and CPSSDRN cohorts were derived from real-world DR screening programs assisted by Phoebusmed. For the ADRS and UDRS datasets, the participants were recruited in regions of Algeria and Uzbekistan, respectively. These fundus images were captured using a variety of desktop and handheld fundus cameras from Canon, Topcon, Carl Zeiss, Optomed and MicroClear.

DR severity was graded into five levels (non-DR, mild nonproliferative DR (NPDR), moderate NPDR, severe NPDR or proliferative DR (PDR), respectively), according to the International Clinical Diabetic Retinopathy Disease Severity Scale (AAO, October 2002)^55^. Diabetic macular edema (DME) was considered to be present when there was retinal thickening at or within one disk diameter of the macular center or definite hard exudates in this region^56^. Referable DR was defined as moderate NPDR or worse, DME or both. The adjudication process and interrater reliability of DR and DME grading of each dataset are presented in Supplementary Table 9. Retinal photographs were flagged as ungradable according to our previous study^24^. Diabetes was diagnosed according to the latest American Diabetes Association guidelines^57^.

### The architecture of the DeepDR-LLM system

The DeepDR-LLM consists of two modules: the LLM module (module I) and the DeepDR-Transformer module (module II). Module II is used for image quality assessment, lesion segmentation and DR/DME grading from standard or portable fundus images, based on image-based DL. Module I is used for integrating clinical metadata of people with diabetes, including medical history, physical examinations, laboratory tests and DR/DME diagnosis results, to provide personalized diabetes management recommendations, based on LLM. Specifically, DR/DME diagnosis results could be derived from medical records or module II. In the integrated fashion, DeepDR-LLM could combine DR/DME diagnosis results derived from module II using fundus images as inputs with other clinical metadata to generate individualized management recommendations for people with diabetes.

#### LLM module’s supervised fine-tuning

Module I is a domain knowledge enhanced LLM model that is designed to formulate diabetes management recommendations, based on various clinical metadata from medical history, physical examinations, laboratory tests, and DR and DME diagnosis results. The primary foundational LLM (that is, LLaMA) was not directly effective in generating diabetes management recommendations due to a lack of domain-specific knowledge. Recognizing this gap, we developed a supervised fine-tuning approach to integrate diabetes management-related knowledge into the LLM training process. This approach could enhance the model’s capability to generate diabetes management recommendations by adding essential domain knowledge to the foundational LLM. The dataset for supervised fine-tuning was retrospectively sourced from 371,763 paired clinical data and real-world management recommendations from 267,730 participants from Shanghai Sixth People’s Hospital and Huadong Sanatorium after deidentification. Characteristics of the dataset are presented in Supplementary Table 10. Our proposed supervised fine-tuning approach can work with various LLM models, and we used LLaMA-7B as the foundational LLM for module I in further experiments.

As updating all parameters (that is, the original weights of the LLM) during the fine-tuning of LLM is evidently not optimal in terms of efficiency^58^, we employ the LoRA^59^ and Adapter^60^ techniques here. Specifically, LoRA adds additional network layers, forming a bypass path adding to the original LLM vertically, which emulates intrinsic rank by executing a one-dimensionality reduction followed by a dimensionality increase. During training, the parameters of LLM remain fixed, with only the matrices *A* (for reduction) and *B* (for expansion) undergoing training. The dimensionality-reducing matrix *A* is initialized with a random Gaussian distribution, whereas the dimensionality-expanding matrix *B* is initialized as a zero matrix. The process is formulated as

$$y={{{W}}}_{{{\it0}}}x+{{BA}}x,$$

where ${x}$ and $y$ are the input and output, respectively. ${{{W}}}_{\it0}$ is the pretrained weight of the original LLM.

Besides, within each Transformer layer of LLM, we embed additional initialized Adapter networks, which are used for dimensionality reduction and subsequent expansion of the Transformer’s feature representations. Each Adapter network, consisting of a two-layered multilayer perceptron (MLP) and an activation layer, is behind the feed-forward layer and before the residual connection in a Transformer layer.

Combining the above two techniques, the training focuses solely on the newly incorporated layers, with the parameters of the original LLM frozen. For the training phase, we set a learning rate of 10^−4^ with a cosine learning rate scheduler, a warmup ratio of 0.03 and training epochs of 10. For the detailed training parameters, we used a batch size of 8, selected mapping dimensions of 4,096 for both LoRA and Adapters, and limited the maximum text length to 512 tokens, with a rank of 64, an alpha of 128 and a dropout rate of 0.05.

#### DeepDR-Transformer module’s development and training

As mentioned before, module II serves as a tool for module I in analyzing fundus images for DR predictions. So, we propose a separate model named DeepDR-Transformer, which can extract distinct features from fundus images after fine-tuning on specific tasks.

We address the prediction and analysis of fundus images, including two main objectives: standard retinal image prediction and portable retinal image prediction. We utilize standard fundus images and related labels from the developmental dataset for model training. Moreover, we incorporate the Vision Transformer (ViT) architecture^61^ and conduct supervised training with this dataset. We train DeepDR-Transformer for four tasks using standard fundus images: quality assessment models for images (determining gradability), DR grading prediction models, prediction models for DME (present or absent) and lesion segmentation models (microaneurysms, hemorrhages, cotton-wool spots (CWS) and hard exudates). For each model, we load pretrained weights from ImageNet^62^, initiating end-to-end fine-tuning thereafter. For the structured prediction output yielded by this module II (DeepDR-Transformer), we devise standardized linguistic templates, for example, ‘DR grade: 0 (DR not present); DME grade: 0 (DME not present)’. These linguistic templates could be subsequently integrated as a part of the input prompt for module I (LLM module), thus forming the integrated DeepDR-LLM system altogether. For instance, the generated DR/DME diagnosis results generated by DeepDR-Transformer, along with other clinical metadata could be fed into the LLM module to generate individualized management recommendations for people with diabetes.

### DeepDR-Transformer fine-tuning for the classification and segmentation from standard fundus images

We choose ViT as the backbone model of our DeepDR-Transformer for its robust performance in modeling images. Our DeepDR-Transformer module is initialized by the pretrained weights from ImageNet and then fine-tuned on the developmental dataset for image quality assessment, DR grading, DME grading and lesion segmentation.

The architecture of the DeepDR-Transformer module is composed of a series of Transformer layers. We represent the output features of these layers as ${Z}^{1},{Z}^{2},\cdots ,{Z}^{n}$, where ${Z}^{n}$ corresponds to the feature derived from the *n*th Transformer layer. Our DeepDR-Transformer model is initialized by the pretrained weights from ImageNet and then fine-tuned on the developmental dataset for four classification and segmentation tasks, respectively.

The tasks of fundus image quality assessment, DR grading and DME grading are three classification problems. We apply the global average pooling to the final layer feature ${Z}^{n}$ of the DeepDR-Transformer module. Subsequently, it is processed by a fully connected linear layer to produce a vector that matches the number of classes in the respective classification task.

The objective of the fundus image lesion segmentation is to generate lesion pixel-level masks within the original two-dimensional image size of $h\times w$, where $h$ represents the height and $w$ denotes the width of the original image. Consequently, we transform the feature ${Z}^{n}\in {{\mathbb{R}}}^{\frac{h\times w}{p\times p}\times c}$ into a feature ${O}_{S}\in {{\mathbb{R}}}^{\frac{h}{p}\times \frac{w}{p}\times c}$, where $p$ is the patch size and $c$ is the number of channels. We alternate between convolutional layers and upsampling operations with a factor of $2\times$. Thus, to restore from ${O}_{{\mathrm{S}}}$ to the original size of the input image, four upsampling operations are required. The final channel number is adjusted to 5, where the 0th channel represents the background and the other channels represent the lesions.

All these tasks are considered classification problems (with segmentation being pixel-level classification). The loss function employed across these tasks is cross-entropy loss. We set the number of Transformer layers $n$ as 12 and the patch size $p$ as 16. We used the standardized structure for Transformer layers, with the following parameters for each layer: an embedding size of 768, an MLP size of 3072 (derived from an MLP ratio of 4) and 12 attention heads. The activation function is Gaussian error linear units, and layer normalization is applied. Our learning strategy includes a learning rate set at ${10}^{-3}$, a weight decay of 0.05 and a layer decay of 0.75. We leverage the stochastic gradient descent optimizer for optimization tasks. To enhance stability and mitigate overfitting, the learning rate is scheduled to decrease by a factor of 0.1 every 10 epochs throughout a span of 40 epochs. Each gradient update iteration is configured with a batch size of 16, and the model’s input image resolution is set at 448 × 448 pixels. To improve the training dataset’s diversity and prevent overfitting, data augmentation techniques are utilized, including random resized cropping, affine transformations, horizontal and vertical flips, and Krizhevsky-inspired color augmentation. This color augmentation method introduces color noise to images based on precomputed eigenvectors and eigenvalues. It generates a color vector from a normal distribution (mean 0, standard deviation 0.5), calculates the noise using these eigenvalues and eigenvectors, and adds the resulting noise to the input image to achieve realistic color variation.

### Transfer learning from standard to portable fundus images

The fine-tuned DeepDR-Transformer models, initially trained on standard fundus images, may yield inconsistent results when deployed on portable fundus images, given the inherent disparities in equipment, noise and image dimensions. To address this, we utilize transfer learning^63^ on portable device images. This adaptation leveraged a tuning set derived from the NDSP dataset, including labels for image quality assessment, DR grading and DME detection.

#### Integration of module I and module II

In our DeepDR-LLM system, there are two modes of integrating module I and module II.

In the physician-involved integration mode, the outputs of module II (that is, fundus image gradability; the lesion segmentation of microaneurysm, CWS, hard exudate and hemorrhage; DR grade; and DME grade) could help physicians generate DR/DME diagnosis results (that is, fundus image gradability; DR grade; DME grade; and the presence of lesions). These DR/DME diagnosis results and other clinical metadata will be fed into module I to generate individualized management recommendations for people with diabetes.

In the automated integration mode, the DR/DME diagnosis results from module II and other clinical metadata could be automatically fed into module I to generate individualized management recommendations for people with diabetes. Specifically, the DR/DME diagnosis results include fundus image gradability, DR grade, DME grade classified by module II, and the presence of lesions segmented out by module II.

### Evaluation of the LLM module in a retrospective dataset

To evaluate the capability of the LLM module to provide comprehensive diabetes management recommendations in both English and Chinese languages, we curated a retrospective dataset comprising 100 cases randomly selected from CNDCS (Supplementary Table 3). The flowchart of the evaluation is depicted in Extended Data Fig. 2.

We first translated the case scenarios into English. An international expert panel was then convened to derive reference evidence-based management recommendations from initial drafts created by four senior consultant-level endocrinologists (W.J., Y.B., H.L. and J.Y.). The international expert panel comprised eight endocrinologists—J.C.N.C., J.B.E.-T., L.C., A.O.Y.L., J.E.S., L.-L.L., R.S. and Y.M.B.—and two ophthalmologists, G.S.W.T. and L.J.C. After thorough review and consensus-building discussions, this group of ten experts subsequently agreed upon the English model answers, establishing the benchmark for the management recommendation evaluations in English.

For the Chinese recommendation evaluations, three consultant-level endocrinologists (W.J., H.L. and J.Y.) and two consultant-level ophthalmologists (T.C. and Q.W.) first translated the English reference recommendations into Chinese. They further contextualized these by incorporating guidelines from the Chinese Diabetes Society^64^, aligning the recommendations with local clinical practices in China. These Chinese model answers, which had gone through careful evaluations by Chinese experts, were then applied for assessments in the Chinese language.

Utilizing the aforementioned 100 cases, we generated management recommendations using both the nontuned LLaMA and our fine-tuned LLM module in DeepDR-LLM, in both English and Chinese. For the English-language assessment, we invited an endocrinology resident and a PCP (A.A., with more than 10 years of clinical experience), to formulate management strategies for these cases. The recommendations from LLaMA, DeepDR-LLM, the resident and the PCP were then anonymized and subsequently appraised by a separate assessment panel of eight consultant-level physicians (L.-L.L., C.C.L., H.C.T., Z.H.L., C.S.-Y.T., S.L.K., A.Y.L.L. and S.F.M.), measured against the preestablished model answers in English described above. In a parallel process for the Chinese-language assessment, we sought recommendations from an endocrinology resident and a PCP (Y. Huang, with 15 years of clinical experience), from China. These recommendations were similarly anonymized and then evaluated by a separate assessment panel of four consultant-level endocrinologists from China, against the Chinese model answers previously generated. The 100 cases were distributed at random for assessment in both English and Chinese. Evaluations were anchored to three domains: the extent of inappropriate content, the extent of missing content and the likelihood of possible harm. This evaluation framework was adapted from a methodology employed in a prior study^31^ (refer to Supplementary Table 4). Supplementary Table 8 shows an example of one case, along with its corresponding model answer for management, and four management recommendations provided by LLaMA, DeepDR-LLM, PCP and endocrinology resident.

Moreover, we have conducted an additional ablation study to investigate whether the integration of the DeepDR-Transformer module, affects the performance of diabetes management recommendations. In our original analysis of the head-to-head comparative analysis of management recommendations provided by DeepDR-LLM, LLaMA, PCPs and endocrinology residents, we did not utilize the DeepDR-Transformer module. We included participants with gradable standard fundus images. We just input the ground truth DR/DME grading and other clinical metadata into the LLM module to generate diabetes management recommendations.

To investigate whether the integration of the DeepDR-Transformer module, an image-based DL module (module II), would affect the performance of diabetes management recommendations, we conducted ablation studies in both English and Chinese languages. The design of the ablation studies is shown in Supplementary Fig. 3. There were three arms in the comparison:

1. Arm 1: input the ground truth DR/DME diagnosis results (that is, fundus image gradability, DR grade, DME grade and the presence of lesions) and other clinical metadata into the LLM module.
2. Arm 2 (using the automated integration mode of the DeepDR-LLM system): input the DR/DME diagnosis results derived from the DeepDR-Transformer module (module II) and other clinical metadata into the LLM module.
3. Arm 3: input the other clinical metadata but without DR/DME diagnosis results into the LLM module.

For evaluations in English, we invited ten physicians from Singapore, Malaysia, Spain and the USA. For evaluations in Chinese, we invited four consultant-level endocrinologists from China. The evaluation results are shown in Supplementary Fig. 4. The results showed that the performance of the LLM module (module I) after integration with module II (that is, arm 2 in this experiment) was comparable to that using the ground truth DR/DME diagnosis results as inputs (arm 1). Expectedly, when DR/DME diagnosis results were not input into the LLM module, the performance of the LLM module was significantly decreased.

### Evaluation of the performance of the DeepDR-Transformer on retrospective datasets

The DeepDR-Transformer module was retrospectively developed and validated in 14 datasets with standard fundus images and 7 datasets with portable fundus images as described before. The characteristics of the participants and eyes used in the performance evaluation of DeepDR-Transformer are summarized in Supplementary Tables 1 and 2.

For image quality assessment, we assessed the discriminative performance of the DeepDR-Transformer module for gradability assessment (gradable or ungradable image) on the internal test dataset, four external test datasets with standard fundus images (NDSP, DRPS, WTHM and PUDM) and six external test datasets with portable fundus images (CPSSDRE, CPSSDRM, CPSSDRW, CPSSDRN, ADRS and UDRS).

For lesion segmentation, we annotated retinal lesions, including microaneurysms, CWS, hard exudates and hemorrhages on 5,690 gradable eyes (11,380 images) in the developmental dataset and 2,438 gradable eyes (4,876 images) in the internal test dataset (7:3). For retinal lesion annotation, each fundus image was annotated by two ophthalmologists. Two ophthalmologists generated two lesion annotations for each type of lesion. We considered the two annotations valid if the Intersection over Union (IoU) between them was greater than 0.85. Otherwise, a senior supervisor would check the annotations and give feedback to provide guidance. The image would be reannotated by the two ophthalmologists until the IoU was larger than 0.85. Finally, we took the union of valid annotations as the final ground truth segmentation annotation. We assessed the performance of DeepDR-Transformer for segmenting microaneurysm, CWS, hard exudate and hemorrhage on the internal test dataset.

For DR grading, we assessed the performance of DeepDR-Transformer for detecting early-to-late stages of DR, DME and referable DR on the internal test dataset and 12 external test datasets with standard fundus images (NDSP, DRPS, WTHM, PUDM, CNDCS, GDES, CUHK-STDR, SEED, SiDRP, SN-DREAMS, TNDRSP and UKB). Moreover, we assessed the performance of DeepDR-Transformer for detecting referable DR on six external test datasets with portable fundus images (CPSSDRE, CPSSDRM, CPSSDRW, CPSSDRN, ADRS and UDRS).

### Evaluation of DeepDR-Transformer as an assistive tool in identifying referable DR

In this retrospective evaluation, we enlisted three distinct study sites: Huadong Sanatorium in the urban area of Shanghai, China; The People’s Hospital of Sixian County in the rural area of Anhui Province, China; and Singapore National Eye Centre, Singapore. While the two study sites in China employed PCPs for DR grading, the Singapore study site utilized professional graders. At two study sites in China, we recruited 6 PCPs with different levels of experience in DR grading from each study site: 2 PCPs under 2 years (junior), 2 PCPs around 4 years (intermediate) and 2 PCPs over 6 years (senior), respectively. At the study site in Singapore, we recruited three graders with varying levels of experience in DR grading: one junior grader with under 2 years of experience, one intermediate grader with 4 years and one senior grader with over 6 years of experience.

For the fundus images used in the study sites in China, 500 gradable eyes of standard fundus images (250 nonreferable eyes and 250 referable eyes) were randomly selected from six external test datasets (NDSP, DRPS, WTHM, PUDM, CNDCS and GDES), while 500 gradable eyes of portable fundus images (250 nonreferable eyes and 250 referable eyes) were randomly selected from six external test datasets (CPSSDRE, CPSSDRM, CPSSDRW, CPSSDRN, ADRS and UDRS). For the fundus images used in the study site in Singapore, 300 gradable eyes of standard fundus images (150 nonreferable eyes and 150 referable eyes) were randomly selected from the SEED study. Referable DR was defined as moderate NPDR or worse, DME or both.

To evaluate the accuracy and time efficiency of detecting referable DR cases, we conducted a comparative analysis before and after the integration of the DeepDR-Transformer module into the grading process. Initially, all human experts (that is, PCPs or professional graders) determined the referability of cases without the aid of DeepDR-Transformer. After a washout period of 1 week to minimize recall bias, these experts reassessed the same cases, this time with the assistance of the DeepDR-Transformer module. To ensure the integrity of the evaluation, the sequence of the cases was randomized before each grading session.

### Real-world prospective study

The real-world two-arm, prospective study was conducted in Huadong Sanatorium (affiliated to Shanghai Municipal Health Commission), which is a public medical institution integrating high-volume primary care and health examinations. The study aimed to investigate the impact of the DeepDR-LLM system on patient health outcomes, and satisfaction of both patients and PCPs, when deployed into a high-volume primary care setting. This real-world prospective study was approved by the Ethics Committee of Huadong Sanatorium (2023-08, approved 2 April 2023). The number of enrolled participants was estimated on the basis of the proportion of participants with diabetes and average visits per week in the study site, before the deployment of DeepDR-LLM.

The study design of the real-world prospective study is shown in Extended Data Fig. 3 (showing numbers of participants included in the outcome analysis), and the flow diagram illustrating the screening, selection, and management of study participants is shown in Supplementary Fig. 2. In these 12 weeks, 20,124 participants attended the health examinations. They received medical history taking, physical examinations, laboratory tests and fundus examinations (Supplementary Table 11). Among them, patients with diabetes and gradable fundus images (*n* = 1,994) were subsequently recruited and included in this study. Details of the inclusion and exclusion criteria are shown in Supplementary Section B. These participants were allocated into two arms (the unassisted PCP arm and the PCP+DeepDR-LLM arm) according to the visit time of the participant. The physician-involved integration mode of the DeepDR-LLM system was deployed in the PCP+DeepDR-LLM arm. Participants attending health examinations from 10 April 2023 to 21 May 2023 (first 6 weeks of evaluation period) were included in the unassisted PCP arm, while those from 22 May 2023 to 2 July 2023 (later 6 weeks of evaluation period) were included in the PCP+DeepDR-LLM arm. In this study, a total of 12 PCPs were responsible for primary diabetes care management (Supplementary Table 12). In the unassisted PCP arm, based on examination results, PCPs gave management recommendations. In the PCP+DeepDR-LLM arm, the DeepDR-LLM system was integrated into the clinical workflow (Extended Data Fig. 4). Initially, PCPs gave management recommendations independently. Then, the DeepDR-LLM system assisted PCPs in generating DR/DME diagnosis results and utilized DR/DME diagnosis results and patient information from the electronic health systems, including medical history, physical examinations and laboratory tests to automatically generate recommendations. Subsequently, PCPs edited and produced their final recommendations by taking DeepDR-LLM’s recommendations into account. In both arms, participants were given treatment advice for diabetes face to face by PCPs based on the above recommendations (details in Supplementary Section B).

These participants registered on the mobile follow-up platform deployed in the study site, which could reach the participants via instant messaging and collect information on their current condition of diabetes management using online questionnaires. They were followed up at 2 weeks and/or 4 weeks through the mobile follow-up platform. For all participants diagnosed as referable DR, they were contacted at the 2-week follow-up to check whether (and when) they attended appointments with an ophthalmologist. For all participants with newly diagnosed diabetes, they filled out a questionnaire investigating their status of diabetes management at baseline, 2-week follow-up and 4-week follow-up (Extended Data Fig. 3). The questionnaire investigated the frequency of blood glucose monitoring, physical therapy, nutrient therapy, drug therapy and cessation of drinking and smoking.

The post-deployment evaluation of management recommendations (ranking, quality and empathy) was conducted in substudies I and II of the PCP+DeepDR-LLM arm, which was provided by three consultant-level endocrinologists and participants. For participants, their opinions on three recommendations were collected at the 4-week follow-up. We collected opinions from 372 participants with newly diagnosed diabetes and/or referable DR (6 participants with both newly diagnosed diabetes and referable DR) in the PCP+DeepDR-LLM arm. Each of the three consultant-level endocrinologists was invited to evaluate all the cases. For each case, the PCP, DeepDR-LLM and PCP+DeepDR-LLM’s recommendations were anonymized and randomly ordered. The endocrinologists and surveyed participants ranked these three recommendations and judged both ‘the quality of information provided’ (very poor, poor, acceptable, good or very good) and ‘the empathy or bedside manner provided’ (not empathetic, slightly empathetic, moderately empathetic, empathetic or very empathetic).

Furthermore, PCPs who used the DeepDR-LLM system in this real-world study were invited to complete a satisfaction questionnaire within two weeks after the conclusion of the study. The questionnaire included seven-item questions assessing these PCPs’ views regarding the integration of DeepDR-LLM into daily routine practice (Extended Data Table 6).

### Statistical analysis

In the retrospective evaluation of the LLM module in both English and Chinese languages, the total score (defined as the sum of domain-specific scores) was calculated by summing the scores gained in three domains, ranging from 3 to 9 points. For ‘extent of inappropriate content’ and ‘extent of missing content’, 1 point was given for ‘Present, substantial clinical significance’, 2 points for ‘Present, little clinical significance’ and 3 points for ‘None’. For ‘likelihood of possible harm’, 1 point was given for ‘High’, 2 points for ‘Medium’ and 3 points for ‘Low’. We compared the total scores of DeepDR-LLM, LLaMA, PCPs and endocrinology residents using the Friedman tests. Post-hoc pairwise comparisons were performed using the Wilcoxon signed-rank test. *P* values for multiple comparisons were adjusted using the Bonferroni method.

In the development and validation of the DeepDR-Transformer module, the performance of the image quality assessment and DR grading was measured by the AUCs generated by plotting sensitivity (true positive rate) versus 1 − specificity (false positive rate). The operating thresholds for sensitivity and specificity were selected using the Youden index. The performance of lesion segmentation was measured using the IoU and *F* score. Cluster-bootstrap, biased-corrected, asymptotic two-sided 95% confidence intervals (CIs) adjusted for clustering by patients were calculated and presented for proportions (sensitivity, specificity) and AUC, respectively^22^.

In the evaluation of DeepDR-Transformer as an assistive tool for PCPs and professional graders in identifying referable DR, the performance was measured by sensitivity and specificity of detecting referable DR. The 95% CIs of the assessment time per eye were calculated using bootstrap methods. The assessment time before and after the DeepDR-Transformer assistance was compared using Wilcoxon signed-rank tests.

In the real-world prospective study, to compare the differences in outcomes at baseline, 2-week and 4-week follow-up among participants with newly diagnosed diabetes or referable DR between two arms, we performed linear mixed models, logistic regression models, and linear regression models, adjusting for age, sex and baseline HbA1c. For post-deployment evaluation of management recommendations by both endocrinologists and participants, we reported the percentage of evaluators for their first-choice preference as well as the Clopper–Pearson 95% CI. All hypotheses tested were two-sided, and a *P* value of less than 0.05 was considered statistically significant.

### Reporting summary

Further information on research design is available in the Nature Portfolio Reporting Summary linked to this article.

## Online content

Any methods, additional references, Nature Portfolio reporting summaries, source data, extended data, supplementary information, acknowledgements, peer review information; details of author contributions and competing interests; and statements of data and code availability are available at 10.1038/s41591-024-03139-8.

## Supplementary information

Supplementary Information Supplementary Figs. 1–4, Tables 1–12 and the study protocol of the real-world prospective study.

Reporting Summary

## Source data

Source Data Main Figures Statistical source data for Figs. 3–5.

## Figures and Tables

**Extended Data Table 1:**
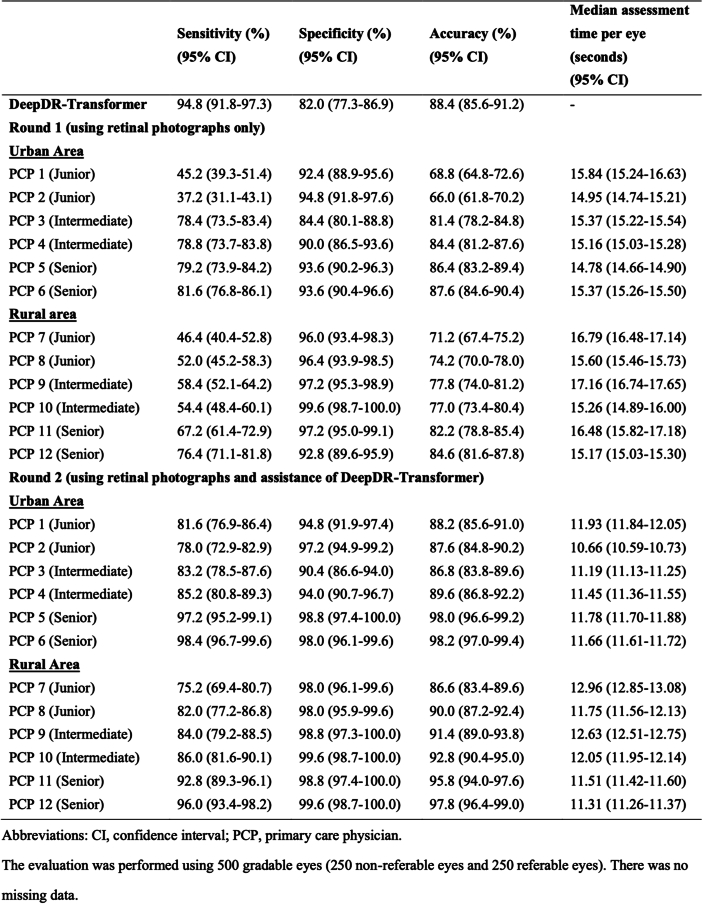
Evaluation of DeepDR-Transformer as an assistive tool for China-based primary care physicians in detecting referable diabetic retinopathy from standard fundus images

**Extended Data Table 2:**
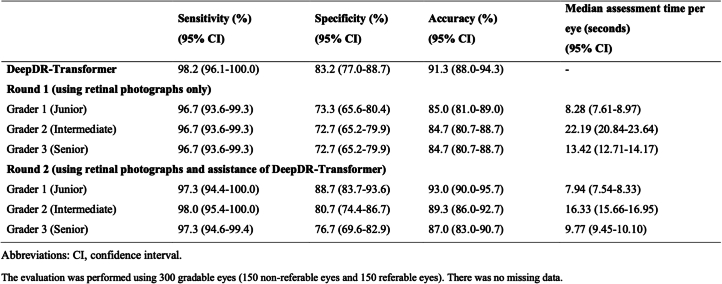
Evaluation of DeepDR-Transformer as an assistive tool for Singapore-based professional graders in detecting referable diabetic retinopathy from standard fundus images

**Extended Data Table 3:**
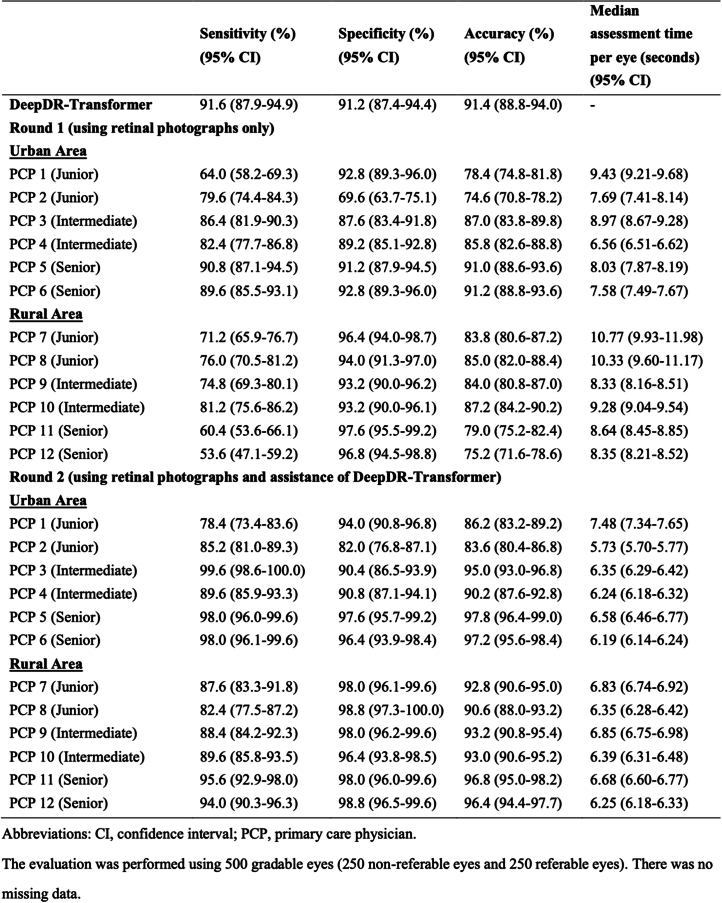
Evaluation of DeepDR-Transformer as an assistive tool for China-based primary care physicians in detecting referable diabetic retinopathy from portable fundus images

**Extended Data Table 4:**
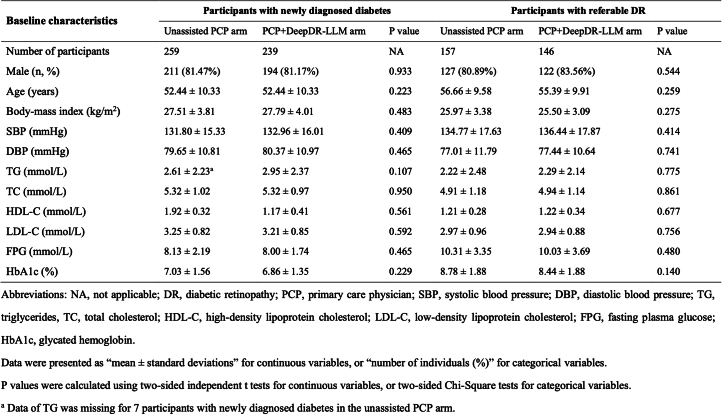
Baseline characteristics of participants with newly diagnosed diabetes or referable diabetic retinopathy in the real-world prospective study, categorized by the unassisted PCP and PCP+DeepDR-LLM arms

**Extended Data Table 5:**
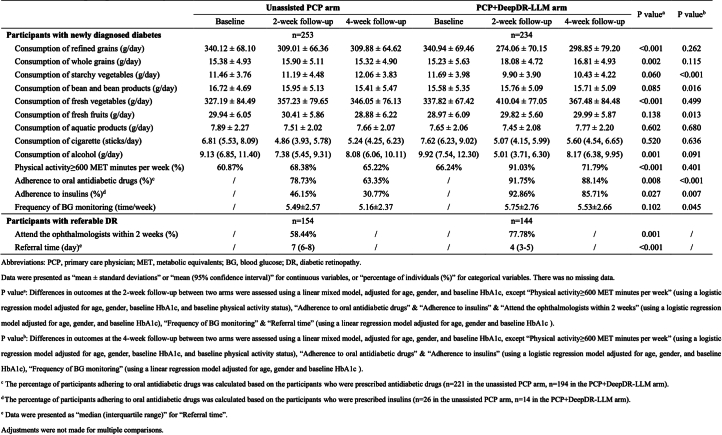
Comparative analysis of self-management behaviors in patients between the unassisted PCP arm and PCP+DeepDR-LLM arm in the real-world prospective study

**Extended Data Table 6:**
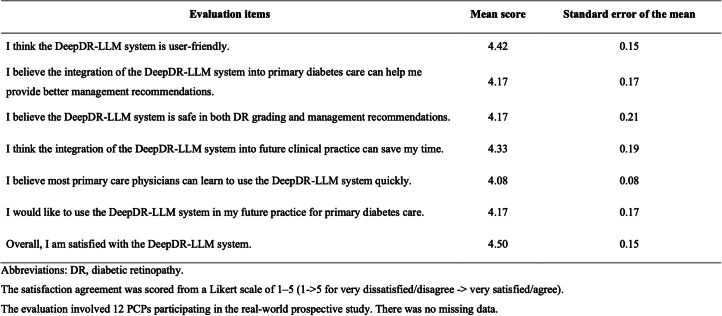
Post-deployment assessment by primary care physicians using the DeepDR-LLM system

## Data Availability

Individual-level patient data can be accessible with the informed consent of the Data Management Committee from institutions and are not publicly available. Interested investigators can obtain and certify the data transfer agreement and submit requests to T.Y.W. (wongtienyin@tsinghua.edu.cn). Investigators who consent to the terms of the data transfer agreement, including, but not limited to, the use of these data only for academic purposes, and to protect the confidentiality of the data and limit the possibility of identification of patients, will be granted access. Requests will be evaluated on a case-by-case basis within one month before receipt of a response. All data shared will be deidentified. For the reproduction of our algorithm code, we have also deposited a minimum dataset at Zenodo (10.5281/zenodo.11501225) (ref. ^65^), which is publicly available for scientific research and noncommercial use. Source data are provided with this paper.

## Code availability

The code being used in the current study for developing the algorithm is provided via GitHub at https://github.com/DeepPros/DeepDR-LLM.
