# Supplementary material for: Integrated image-based deep learning and language models for primary diabetes care
Source: Nat Med. 2024 Jul 19;30(10):2886–96. doi: 10.1038/s41591-024-03139-8 (PMC11485246; doi:10.1038/s41591-024-03139-8)
Supplement: Supplementary file 1 — Supplementary Figs. 1–4, Tables 1–12 and the study protocol of the real-world prospective study. [file 41591_2024_3139_MOESM1_ESM.pdf]

nature medicine

Supplementary information https://doi.org/10.1038/s41591-024-03139-8

Integrated image-based deep learning and language models for primary diabetes care

In the format provided by the authors and unedited

Contents

Section A. Supplementary Figures and Tables

Supplementary Fig. 1. Receiver operating characteristic curve showing performance of DeepDR-Transformer and humans in the evaluation of DeepDR-Transformer in assisting the identification of referable DR in Singapore

Supplementary Fig. 2. Flow diagram illustrating the screening, selection, and management of study participants in the prospective real-world study

Supplementary Fig. 3. Design of the ablation study

Supplementary Fig. 4. Results of the ablation study in both English and Chinese languages

Supplementary Table 1. Summary of the datasets with standard fundus images

Supplementary Table 2. Summary of the datasets with portable fundus images

Supplementary Table 3. Characteristics of participants with diabetes in the retrospective evaluation of the LLM module of the DeepDR-LLM system

Supplementary Table 4. Summary of the different domains along which clinicians evaluate the management recommendations in the retrospective analysis

Supplementary Table 5. Discriminative performance of image quality assessment (gradable or ungradable image) on standard and portable fundus images

Supplementary Table 6. Performance of lesion segmentation on standard fundus images in the internal test set

Supplementary Table 7. Performance of DR grading from standard fundus images and portable fundus images

Supplementary Table 8. Example of management recommendations to one case in the retrospective evaluation of the LLM module

Supplementary Table 9. Adjudication process and inter-rater reliability of DR and DME grading

Supplementary Table 10. Characteristics of the paired clinical data and real-world health management recommendations included in the dataset for supervised fine-tuning of the LLM module

Supplementary Table 11. Summary of laboratory tests and fundus examinations in the real-world prospective study

Supplementary Table 12. Characteristics of the primary care physicians involved in the real-world prospective study

Section B. Study Protocol of the Real-world Prospective Study

Section A. Supplementary Figures and Tables

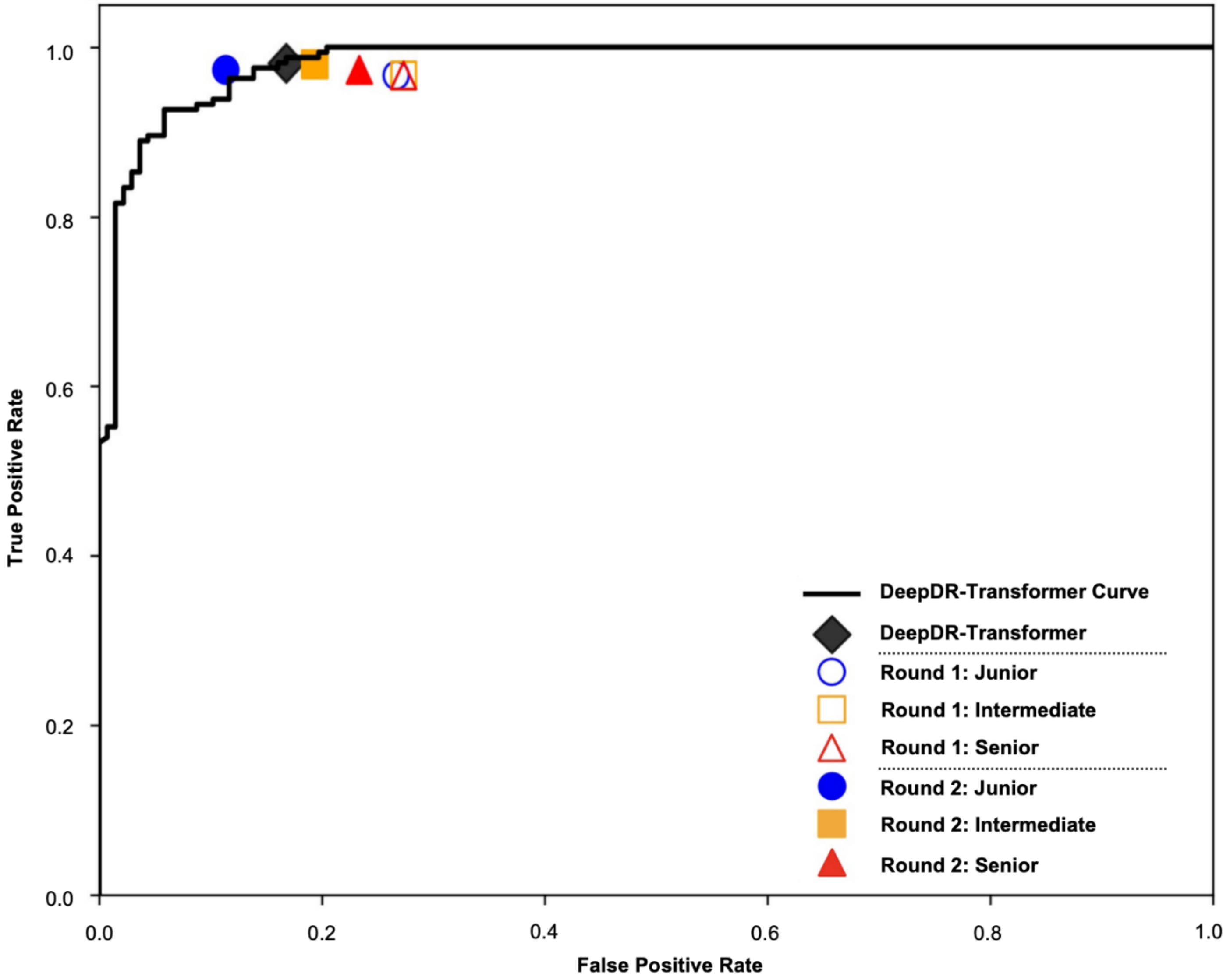

Supplementary Figure 1. Receiver operating characteristic curve showing performance of DeepDR-Transformer and humans in the evaluation of DeepDR-Transformer in assisting the identification of referable DR in Singapore.

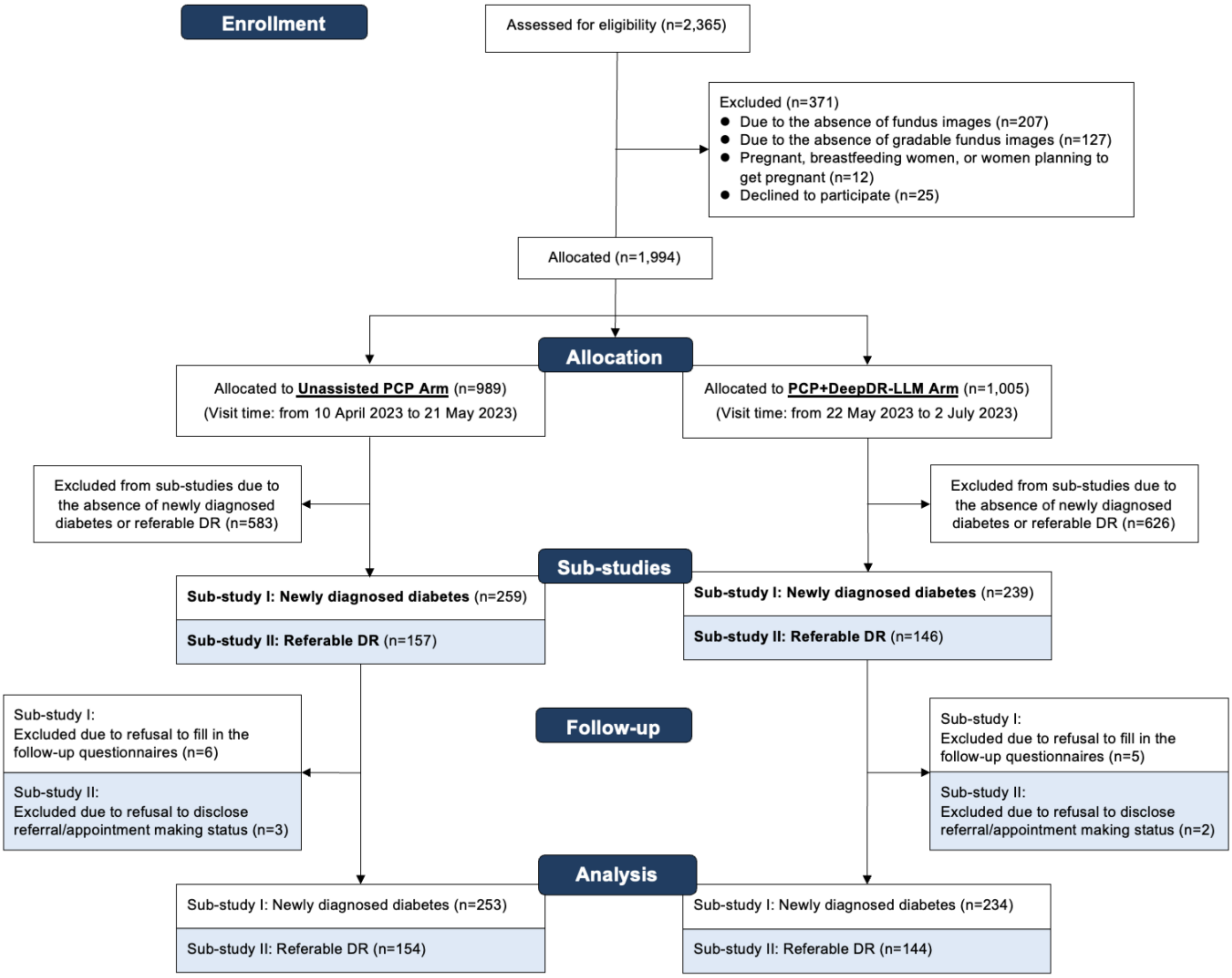

Supplementary Figure 2. Flow diagram illustrating the screening, selection, and management of study participants in the prospective real-world study.

In the outcome analysis: For the unassisted PCP arm, there were a total of 397 participants with diabetes (10 participants with both newly diagnosed diabetes and referable DR); For the PCP+DeepDR-LLM arm, there were a total of 372 participants with diabetes (6 participants with both newly diagnosed diabetes and referable DR).

Abbreviations: PCP, primary care physician; DR, diabetic retinopathy.

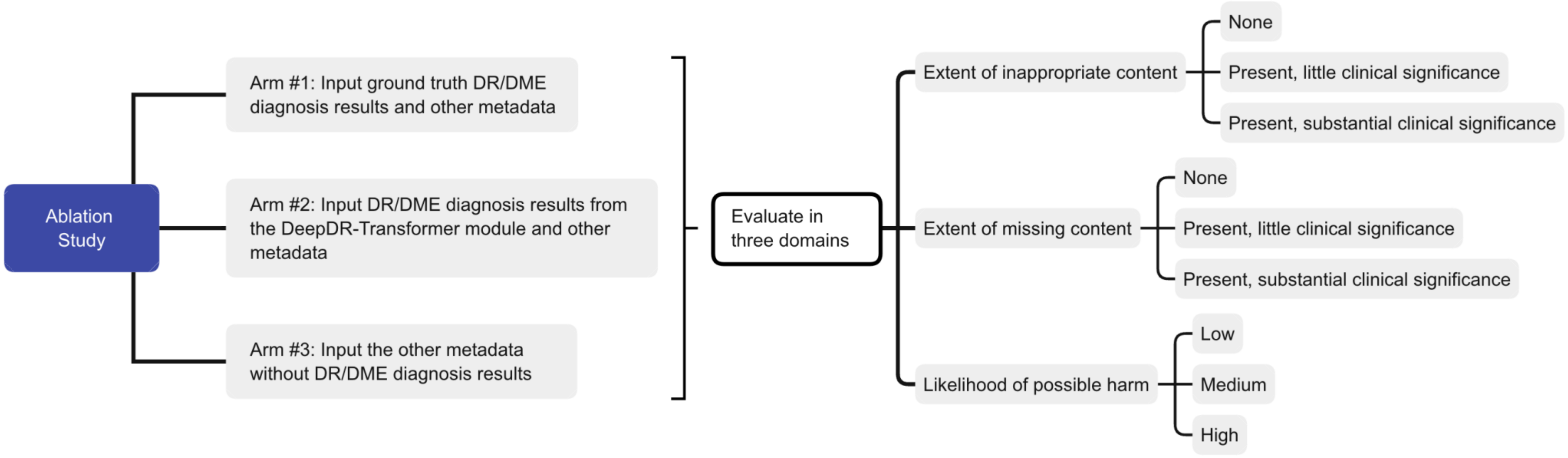

Supplementary Figure 3. Design of the ablation study.

Abbreviations: DR, diabetic retinopathy; DME, diabetic macular edema.

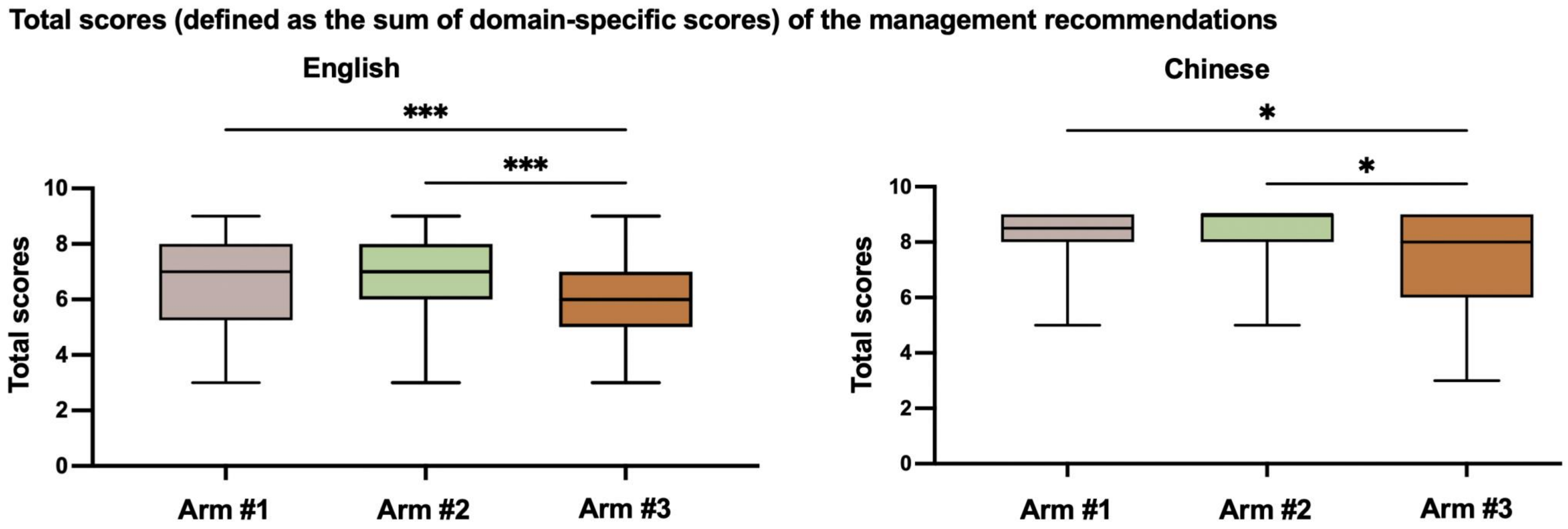

Supplementary Figure 4. Results of the ablation study in both English and Chinese languages.

This figure shows the distribution of the total scores of management recommendations (defined as the sum of domain-specific scores) generated in Arm #1, Arm #2, and Arm #3. Box plot (n=100), median and quartiles; whiskers, data range. The comparison was performed using two-sided Friedman tests. Post-hoc pairwise comparisons were performed using two-sided Wilcoxon signed rank test. P values for multiple comparisons were adjusted using the Bonferroni method. **P*=0.030; ****P*<0.001.

Supplementary Table 1. Summary of the datasets with standard fundus images.

	Developmental dataset	Internal test dataset	External test datasets (n=12)											
	**SIM + SDPP**	**SIM + SDPP**	**NDSP**	**DRPS**	**WTHM**	**PUDM**	**CNDCS**	**GDES**	**CUHK-STDR**	**SEED**	**SiDRP**	**SN-DREAMS**	**TNDRSP**	**UKB**
**Dataset Characteristics**														
Number of participants	128,151	54,922	1,898	22,463	723	365	50,527	2,305	625	2,877	3,280	1,561	6,776	3,346
Number of eyes	256,302	109,844	3,796	44,926	1,446	730	96,756	4,610	1,216	5,754	6,560	3,077	12,438	5,900
Number of images	512,604	219,688	7,592	89,852	2,892	1,460	193,512	9,220	2,432	11,508	13,120	3,077	12,438	5,900
Race or ethnicity	Chinese	Chinese	Chinese	Chinese	Chinese	Chinese	Chinese	Chinese	Chinese	Chinese (20.19%)<br>Indian (44.77%)<br>Malay (35.04%)	Chinese (72.13%)<br>Indian (7.87%)<br>Malay (15.00%)<br>Other (5.00%)	Indian	Thai	White (79.05%)<br>Mixed (0.96%)<br>Asian (9.23%)<br>Black (5.74%)<br>Chinese (0.54%)<br>Other (4.48%)
Male, n (%)	59,684 (46.57%)	25,410 (46.27%)	739 (38.94%)	18,902 (84.15%)	576 (79.67%)	218 (59.73%)	25,112 (49.70%)	986 (42.80%)	339 (54.24%)	1,457 (50.64%)	1,659 (50.58%)	818 (52.40%)	2,129 (31.42%)	2,079 (62.13%)
**Image Characteristics**														
Ungradable images, n (%)	56,232 (10.97%)	23,880 (10.87%)	702 (9.25%)	8,385 (9.33%)	312 (10.79%)	139 (9.52%)	NA	NA	NA	NA	NA	NA	NA	NA
**Eye Characteristics**														
Gradable eyes, n (%)	213,184 (83.18%)	96,247 (87.62%)	3,226 (84.98%)	38,200 (85.03%)	1,217 (84.16%)	638 (87.40%)	96,756 (100%)	4,610 (100%)	1,216 (100%)	5,754 (100%)	6,560 (100%)	3,077 (100%)	12,438 (100%)	5,900 (100%)
DR severity*														
Non-DR	187,036 (87.73%)	84,438 (87.73%)	2,998 (92.93%)	34,104 (89.28%)	1,147 (94.25%)	573 (89.81%)	85,321 (88.18%)	4,024 (87.29%)	544 (44.74%)	4,660 (80.99%)	6,334 (96.55%)	2,659 (86.42%)	10,063 (80.91%)	5,263 (89.20%)
Mild NPDR	7,645 (3.59%)	3,455 (3.59%)	119 (3.69%)	1,160 (3.04%)	26 (2.14%)	17 (2.66%)	2,473 (2.56%)	187 (4.06%)	44 (3.62%)	533 (9.26%)	60 (0.91%)	220 (7.15%)	943 (7.58%)	163 (2.76%)
Moderate NPDR	15,647 (7.34%)	7,064 (7.34%)	100 (3.10%)	1,719 (4.50%)	32 (2.63%)	30 (4.70%)	6,866 (7.10%)	338 (7.33%)	560 (46.05%)	314 (5.46%)	145 (2.21%)	129 (4.19%)	1,173 (9.43%)	353 (5.98%)
Severe NPDR	2,281 (1.07%)	1,035 (1.08%)	3 (0.09%)	890 (2.33%)	9 (0.74%)	17 (2.66%)	1,769 (1.83%)	49 (1.06%)	58 (4.77%)	25 (0.43%)	13 (0.20%)	31 (1.01%)	108 (0.87%)	34 (0.57%)
PDR	575 (0.27%)	255 (0.26%)	6 (0.19%)	327 (0.86%)	3 (0.25%)	1 (0.16%)	327 (0.34%)	12 (0.26%)	10 (0.82%)	222 (3.86%)	8 (0.12%)	38 (1.23%)	151 (1.21%)	87 (1.47%)
DME*	1,236 (0.58%)	558 (0.58%)	24 (0.74%)	278 (0.73%)	10 (0.82%)	4 (0.63%)	490 (0.51%)	27 (0.59%)	199 (16.37%)	142 (2.47%)	130 (1.98%)	75 (2.44%)	898 (7.22%)	105 (1.78%)
Referable DR*	18,526 (8.69%)	8,372 (8.70%)	113 (3.50%)	2,957 (7.74%)	44 (3.62%)	48 (7.52%)	9,002 (9.30%)	401 (8.70%)	632 (51.97%)	580 (10.08%)	166 (2.53%)	214 (6.95%)	1,512 (12.16%)	475 (8.05%)

Abbreviations: SIM, Shanghai Integration Model; SDPP, Shanghai Diabetes Prevention Program; NDSP, Nicheng Diabetes Screening Project; DRPS, Diabetic Retinopathy Progression Study; WTHM, Wuhan Tongji Health Management; PUDM, Peking Union Diabetes Management; CNDCS, China National Diabetic Complications Study; GDES, Guangzhou Diabetic Eye Study; CUHK-STDR, Chinese University of Hong Kong-Sight-Threatening Diabetic Retinopathy; SEED, Singapore Epidemiology of Eye Diseases; SiDRP, Singapore National Diabetic Retinopathy Screening Program; SN-DREAMS, Sankara Nethralaya-Diabetic Retinopathy Epidemiology and Molecular Genetics Study; TNDRSP, Thai National Diabetic Retinopathy Screening Program; UKB, United Kingdom Biobank; DR, diabetic retinopathy; NPDR, non-proliferative diabetic retinopathy; PDR, proliferative diabetic retinopathy; DME, diabetic macular edema, NA, not available.

Data are presented as “number of individuals (%)” for categorical variables.

* DR severity, DME, and referable DR were analyzed on gradable eyes. If any image of one eye was ungradable, the eye was deemed as “ungradable”.

Supplementary Table 2. Summary of the datasets with portable fundus images.

	Fine-tuning dataset	External test datasets (n=6)					
	**NDSP**	**CPSSDRE**	**CPSSDRM**	**CPSSDRW**	**CPSSDRN**	**ADRS**	**UDRS**
**Dataset Characteristics**							
Number of participants	1,576	49,094	18,152	9,014	456	1,004	1,624
Number of eyes	3,152	98,188	36,304	18,028	912	2,008	3,248
Number of images	3,152	98,188	36,304	18,028	912	2,008	3,248
Race or ethnicity	Chinese	Chinese	Chinese	Chinese	Chinese	Algerian	Uzbek
Male, n (%)	616 (39.09%)	20,865 (42.50%)	7,784 (42.88%)	3,797 (42.12%)	191 (41.89%)	NA	NA
**Image Characteristics**							
Ungradable images, n (%)	471 (14.94%)	12,557 (12.79%)	5,406 (14.89%)	2,425 (13.45%)	117 (12.83%)	276 (13.75%)	467 (14.38%)
**Eye Characteristics**							
Gradable eyes, n (%)	2,681 (85.06%)	85,631 (87.21%)	30,898 (85.11%)	15,603 (86.55%)	795 (87.17%)	1,732 (86.25%)	2,781 (85.62%)
DR severity*							
Non-DR	2,473 (92.24%)	73,949 (86.36%)	27,294 (88.34%)	13,565 (86.94%)	691 (86.92%)	1,478 (85.33%)	2,352 (84.57%)
Mild NPDR	117 (4.36%)	3,310 (3.87%)	1,009 (3.27%)	575 (3.69%)	6 (0.75%)	84 (4.85%)	165 (5.93%)
Moderate NPDR	82 (3.06%)	6,724 (7.85%)	2,193 (7.10%)	1,127 (7.22%)	69 (8.68%)	128 (7.39%)	213 (7.66%)
Severe NPDR	3 (0.11%)	1,225 (1.43%)	298 (0.96%)	216 (1.38%)	19 (2.39%)	27 (1.56%)	31 (1.11%)
PDR	6 (0.22%)	423 (0.49%)	104 (0.34%)	120 (0.77%)	10 (1.26%)	15 (0.87%)	20 (0.72%)
DME*	23 (0.86%)	627 (0.73%)	248 (0.80%)	108 (0.69%)	6 (0.75%)	14 (0.81%)	23 (0.83%)
Referable DR*	93 (3.47%)	8,376 (9.78%)	2,597 (8.41%)	1,467 (9.40%)	98 (12.33%)	170 (9.82%)	264 (9.49%)

Abbreviations: NDSP, Nicheng Diabetes Screening Program; CPSSDRE, Chinese Portable Screening Study for Diabetic Retinopathy-East; CPSSDRM, Chinese Portable Screening Study for Diabetic Retinopathy-Middle; CPSSDRW, Chinese Portable Screening Study for Diabetic Retinopathy-West; CPSSDRN, Chinese Portable Screening Study for Diabetic Retinopathy-Northeast; ADRS, Algerian Diabetic Retinopathy Study; UDRS, Uzbek Diabetic Retinopathy Study; DR, diabetic retinopathy; NPDR, non-proliferative diabetic retinopathy; PDR, proliferative diabetic retinopathy; DME, diabetic macular edema; NA, not available.

Data were presented as “mean ± standard deviations (SD)” for continuous variables or “number of individuals (%)” for categorical variables.

*DR severity, DME, and referable DR were analyzed on gradable eyes.

Supplementary Table 3. Characteristics of participants with diabetes in the retrospective evaluation of the LLM module of the DeepDR-LLM system.

Baseline characteristics	Participants randomly selected from CNDCS
Number of participants	100
Age (years)	56.30 ± 9.96
Sex (male: female)	52:48
Current smoker (n, %)	43 (43.00%)
Current drinker (n, %)	59 (59.00%)
Body-mass index (kg/m^2)	26.24 ± 3.08
SBP (mmHg)	135.69 ± 16.40
DBP (mmHg)	78.32 ± 10.54
Heart rate (beats per minute)	79.20 ± 10.13
TG (mmol/L)	2.19 ± 2.39
TC (mmol/L)	4.86 ± 1.17
LDL-C (mmol/L)	2.96 ± 0.91
HDL-C (mmol/L)	1.16 ± 0.26
HbA1c (%)	7.13 ± 1.50
FPG (mmol/L)	8.07 ± 2.19
Estimated glomerular filtration rate (mL/min/1.73 m^2)	90.11 ± 20.54
Urine albumin-to-creatinine ratio (mg/g)	16.34 (5.60, 44.65)
Aspartate aminotransferase (U/L)	19.80 (16.38, 24.43)
Alanine aminotransferase (U/L)	20.20 (15.25, 30.63)
Gamma-glutamyl transferase (U/L)	25.00 (19.00, 38.75)
Participants with referable DR	18 (18.00%)

Abbreviations: CNDCS, China National Diabetes Complications Study; SBP, systolic blood pressure; DBP, diastolic blood pressure; TG, triglycerides, TC, total cholesterol; LDL-C, low-density lipoprotein cholesterol; HDL-C, high-density lipoprotein cholesterol; HbA1c, glycated hemoglobin; FPG, fasting plasma glucose; DR, diabetic retinopathy.

Data were presented as “mean ± standard deviations” or “median (interquartile range)” for continuous variables, or “number of individuals (%)” for categorical variables.

Supplementary Table 4. Summary of the different domains along which clinicians evaluate the management recommendations in the retrospective analysis.

Domains	Questions	Choice
**Extent of inappropriate content**	Does the management recommendation contain any content it shouldn't?	- None<br>- Present, little clinical significance<br>- Present, substantial clinical significance
**Extent of missing content**	Does the management recommendation omit any content it shouldn't?	- None<br>- Present, little clinical significance<br>- Present, substantial clinical significance
**Likelihood of possible harm**	What is the likelihood of possible harm?	- Low<br>- Medium<br>- High

Supplementary Table 5. Discriminative performance of image quality assessment (gradable or ungradable image) on standard and portable fundus images.

Dataset	AUC (95% CI)	Sensitivity (95% CI)	Specificity (95% CI)
**Datasets with standard fundus images**			
**Internal test dataset**	0.917 (0.915-0.919)	0.918 (0.914-0.921)	0.916 (0.915-0.917)
**NDSP**	0.923 (0.913-0.934)	0.925 (0.905-0.944)	0.921 (0.915-0.927)
**DRPS**	0.922 (0.919-0.925)	0.924 (0.919-0.929)	0.920 (0.918-0.922)
**WTHM**	0.919 (0.903-0.934)	0.923 (0.893-0.951)	0.914 (0.903-0.925)
**PUDM**	0.916 (0.892-0.937)	0.914 (0.869-0.955)	0.918 (0.903-0.933)
**Datasets with portable fundus images**			
**CPSSDRE**	0.915 (0.913-0.918)	0.916 (0.911-0.921)	0.915 (0.913-0.917)
**CPSSDRM**	0.915 (0.911-0.919)	0.916 (0.909-0.923)	0.914 (0.911-0.917)
**CPSSDRW**	0.913 (0.907-0.919)	0.914 (0.902-0.924)	0.913 (0.908-0.917)
**CPSSDRN**	0.891 (0.858-0.922)	0.863 (0.798-0.920)	0.919 (0.900-0.938)
**ADRS**	0.924 (0.900-0.946)	0.932 (0.888-0.974)	0.916 (0.897-0.936)
**UDRS**	0.925 (0.911-0.936)	0.927 (0.902-0.948)	0.922 (0.912-0.931)

Abbreviations: AUC, area under the receiver operating characteristic curve; CI, confidence interval; NDSP, Nicheng Diabetes Screening Project; DRPS, Diabetic Retinopathy Progression Study; WTHM, Wuhan Tongji Health Management; PUDM, Peking Union Diabetes Management; CPSSDRE, Chinese Portable Screening Study for Diabetic Retinopathy-East; CPSSDRM, Chinese Portable Screening Study for Diabetic Retinopathy-Middle; CPSSDRW, Chinese Portable Screening Study for Diabetic Retinopathy-West; CPSSDRN, Chinese Portable Screening Study for Diabetic Retinopathy-Northeast; ADRS, Algerian Diabetic Retinopathy Study; UDRS, Uzbek Diabetic Retinopathy Study.

Supplementary Table 6. Performance of lesion segmentation on standard fundus images in the internal test set.

Dataset	Lesion	Interception over union (IoU)	F-score
**Internal test dataset**	Microaneurysm	-	0.803
	Cotton-wool spot	0.734	-
	Hard exudate	0.860	-
	Hemorrhage	0.740	-

For lesion segmentation, the IoU and F-score were calculated based on image-level annotations.

The evaluation of the performance of lesion segmentation was performed using 4,876 fundus images from 2,438 gradable eyes in the internal test dataset.

Supplementary Table 7. Performance of DR grading from standard fundus images and portable fundus images.

Dataset	DR severity	AUC (95% CI)	Sensitivity (95% CI)	Specificity (95% CI)
**Datasets with standard fundus images**				
**Internal test dataset**	Non-DR	0.918 (0.916-0.920)	0.871 (0.869-0.874)	0.965 (0.961-0.968)
	Mild NPDR	0.922 (0.917-0.927)	0.876 (0.865-0.886)	0.968 (0.967-0.969)
	Moderate NPDR	0.919 (0.915-0.923)	0.869 (0.861-0.877)	0.969 (0.967-0.970)
	Severe NPDR	0.923 (0.912-0.933)	0.878 (0.856-0.899)	0.968 (0.967-0.969)
	PDR	0.903 (0.878-0.923)	0.839 (0.789-0.880)	0.967 (0.966-0.968)
	DME	0.913 (0.901-0.926)	0.905 (0.880-0.930)	0.921 (0.919-0.922)
	Referable DR	0.920 (0.917-0.923)	0.919 (0.913-0.925)	0.920 (0.919-0.922)
**NDSP**	Non-DR	0.897 (0.881-0.912)	0.843 (0.830-0.856)	0.952 (0.924-0.976)
	Mild NPDR	0.872 (0.832-0.907)	0.790 (0.711-0.861)	0.954 (0.946-0.961)
	Moderate NPDR	0.913 (0.878-0.947)	0.860 (0.791-0.928)	0.966 (0.959-0.972)
	Severe NPDR	0.815 (0.480-0.984)	0.667 (0.000-1.000)	0.963 (0.956-0.969)
	PDR	0.896 (0.727-0.982)	0.833 (0.500-1.000)	0.959 (0.952-0.966)
	DME	0.909 (0.845-0.953)	0.917 (0.789-1.000)	0.902 (0.892-0.912)
	Referable DR	0.892 (0.860-0.922)	0.885 (0.820-0.945)	0.900 (0.888-0.911)
**DRPS**	Non-DR	0.899 (0.896-0.903)	0.839 (0.835-0.843)	0.960 (0.954-0.966)
	Mild NPDR	0.897 (0.887-0.908)	0.835 (0.816-0.858)	0.960 (0.957-0.962)
	Moderate NPDR	0.902 (0.894-0.911)	0.845 (0.827-0.863)	0.960 (0.958-0.962)
	Severe NPDR	0.902 (0.890-0.913)	0.843 (0.818-0.866)	0.961 (0.959-0.963)
	PDR	0.889 (0.868-0.909)	0.820 (0.777-0.859)	0.959 (0.957-0.961)
	DME	0.917 (0.901-0.933)	0.921 (0.887-0.953)	0.914 (0.911-0.916)
	Referable DR	0.923 (0.918-0.928)	0.922 (0.912-0.931)	0.925 (0.923-0.928)
**WTHM**	Non-DR	0.912 (0.892-0.928)	0.839 (0.817-0.860)	0.986 (0.949-1.000)
	Mild NPDR	0.884 (0.796-0.958)	0.808 (0.630-0.955)	0.961 (0.950-0.971)
	Moderate NPDR	0.919 (0.853-0.978)	0.875 (0.744-1.000)	0.962 (0.951-0.973)
	Severe NPDR	0.923 (0.793-0.983)	0.889 (0.625-1.000)	0.958 (0.945-0.969)
	PDR	0.812 (0.476-0.983)	0.667 (0.000-1.000)	0.957 (0.945-0.969)
	DME	0.956 (0.947-0.964)	1.000 (1.000-1.000)	0.911 (0.893-0.927)
	Referable DR	0.933 (0.892-0.968)	0.932 (0.848-1.000)	0.934 (0.920-0.949)
**PUDM**	Non-DR	0.894 (0.859-0.925)	0.850 (0.819-0.878)	0.938 (0.877-0.987)
	Mild NPDR	0.923 (0.837-0.986)	0.882 (0.714-1.000)	0.965 (0.949-0.979)
	Moderate NPDR	0.904 (0.824-0.967)	0.833 (0.667-0.960)	0.975 (0.962-0.987)
	Severe NPDR	0.857 (0.741-0.946)	0.765 (0.533-0.944)	0.948 (0.929-0.965)
	PDR	0.981 (0.973-0.988)	1.000 (1.000-1.000)	0.962 (0.947-0.976)
	DME	0.955 (0.944-0.965)	1.000 (1.000-1.000)	0.910 (0.888-0.931)
	Referable DR	0.933 (0.891-0.967)	0.917 (0.837-0.981)	0.949 (0.931-0.966)
**CNDCS**	Non-DR	0.899 (0.897-0.901)	0.839 (0.836-0.841)	0.960 (0.956-0.964)
	Mild NPDR	0.901 (0.894-0.908)	0.842 (0.828-0.857)	0.959 (0.958-0.961)
	Moderate NPDR	0.899 (0.895-0.904)	0.838 (0.830-0.847)	0.961 (0.959-0.962)
	Severe NPDR	0.903 (0.895-0.911)	0.847 (0.830-0.862)	0.960 (0.958-0.961)
	PDR	0.905 (0.884-0.924)	0.850 (0.810-0.888)	0.959 (0.958-0.961)
	DME	0.905 (0.892-0.919)	0.896 (0.869-0.922)	0.915 (0.913-0.916)

	Referable DR	0.925 (0.922-0.928)	0.924 (0.918-0.929)	0.926 (0.924-0.927)
**GDES**	Non-DR	0.901 (0.891-0.910)	0.841 (0.829-0.852)	0.961 (0.946-0.975)
	Mild NPDR	0.920 (0.896-0.944)	0.877 (0.830-0.925)	0.963 (0.957-0.968)
	Moderate NPDR	0.889 (0.868-0.911)	0.820 (0.778-0.861)	0.959 (0.953-0.965)
	Severe NPDR	0.949 (0.912-0.980)	0.939 (0.865-1.000)	0.960 (0.954-0.965)
	PDR	0.855 (0.729-0.979)	0.750 (0.500-1.000)	0.960 (0.954-0.965)
	DME	0.941 (0.896-0.962)	0.963 (0.870-1.000)	0.920 (0.912-0.928)
	Referable DR	0.918 (0.902-0.931)	0.913 (0.885-0.938)	0.923 (0.914-0.931)
**CUHK-STDR**	Non-DR	0.853 (0.832-0.872)	0.779 (0.744-0.813)	0.926 (0.908-0.945)
	Mild NPDR	0.819 (0.746-0.888)	0.705 (0.559-0.837)	0.934 (0.920-0.949)
	Moderate NPDR	0.840 (0.818-0.860)	0.736 (0.699-0.773)	0.944 (0.924-0.960)
	Severe NPDR	0.807 (0.742-0.868)	0.672 (0.545-0.795)	0.942 (0.928-0.956)
	PDR	0.921 (0.795-0.976)	0.900 (0.636-1.000)	0.942 (0.928-0.955)
	DME	0.890 (0.866-0.914)	0.899 (0.857-0.941)	0.880 (0.860-0.899)
	Referable DR	0.905 (0.888-0.922)	0.894 (0.868-0.917)	0.916 (0.893-0.938)
**SEED**	Non-DR	0.842 (0.833-0.851)	0.751 (0.738-0.762)	0.933 (0.918-0.947)
	Mild NPDR	0.834 (0.814-0.854)	0.734 (0.694-0.774)	0.935 (0.928-0.942)
	Moderate NPDR	0.859 (0.835-0.882)	0.777 (0.728-0.822)	0.941 (0.935-0.947)
	Severe NPDR	0.869 (0.776-0.945)	0.800 (0.611-0.952)	0.937 (0.931-0.944)
	PDR	0.845 (0.815-0.873)	0.752 (0.694-0.807)	0.938 (0.931-0.944)
	DME	0.918 (0.899-0.936)	0.944 (0.904-0.979)	0.892 (0.884-0.900)
	Referable DR	0.906 (0.894-0.919)	0.912 (0.887-0.935)	0.901 (0.893-0.909)
**SiDRP**	Non-DR	0.837 (0.818-0.856)	0.750 (0.738-0.761)	0.925 (0.887-0.959)
	Mild NPDR	0.809 (0.746-0.869)	0.683 (0.557-0.803)	0.935 (0.929-0.941)
	Moderate NPDR	0.851 (0.814-0.884)	0.759 (0.686-0.825)	0.943 (0.937-0.949)
	Severe NPDR	0.775 (0.634-0.905)	0.615 (0.333-0.875)	0.935 (0.929-0.941)
	PDR	0.781 (0.592-0.968)	0.625 (0.250-1.000)	0.937 (0.930-0.943)
	DME	0.870 (0.839-0.898)	0.846 (0.784-0.903)	0.894 (0.886-0.902)
	Referable DR	0.895 (0.869-0.919)	0.880 (0.828-0.927)	0.910 (0.904-0.918)
**SN-DREAMS**	Non-DR	0.850 (0.837-0.863)	0.748 (0.731-0.764)	0.952 (0.931-0.972)
	Mild NPDR	0.837 (0.810-0.866)	0.736 (0.682-0.794)	0.938 (0.930-0.947)
	Moderate NPDR	0.823 (0.783-0.861)	0.721 (0.643-0.794)	0.926 (0.916-0.936)
	Severe NPDR	0.778 (0.685-0.866)	0.613 (0.429-0.792)	0.943 (0.934-0.951)
	PDR	0.836 (0.766-0.907)	0.737 (0.594-0.878)	0.935 (0.926-0.944)
	DME	0.891 (0.859-0.925)	0.893 (0.829-0.959)	0.888 (0.877-0.900)
	Referable DR	0.894 (0.870-0.917)	0.874 (0.828-0.918)	0.914 (0.903-0.924)
**TNDRSP**	Non-DR	0.843 (0.836-0.849)	0.755 (0.746-0.764)	0.931 (0.921-0.942)
	Mild NPDR	0.843 (0.829-0.857)	0.748 (0.719-0.775)	0.939 (0.935-0.944)
	Moderate NPDR	0.840 (0.826-0.852)	0.739 (0.712-0.764)	0.940 (0.935-0.944)
	Severe NPDR	0.853 (0.814-0.894)	0.769 (0.691-0.849)	0.938 (0.934-0.942)
	PDR	0.856 (0.823-0.890)	0.775 (0.709-0.841)	0.938 (0.933-0.942)
	DME	0.885 (0.874-0.895)	0.883 (0.863-0.904)	0.888 (0.882-0.893)
	Referable DR	0.914 (0.906-0.921)	0.917 (0.902-0.930)	0.911 (0.906-0.917)
**UKB**	Non-DR	0.899 (0.890-0.908)	0.838 (0.827-0.849)	0.961 (0.945-0.975)
	Mild NPDR	0.895 (0.864-0.923)	0.828 (0.767-0.883)	0.962 (0.958-0.967)

	Moderate NPDR	0.911 (0.891-0.929)	0.864 (0.825-0.900)	0.958 (0.952-0.963)
	Severe NPDR	0.832 (0.754-0.906)	0.706 (0.548-0.853)	0.959 (0.953-0.964)
	PDR	0.917 (0.880-0.948)	0.874 (0.800-0.935)	0.960 (0.955-0.965)
	DME	0.892 (0.855-0.924)	0.867 (0.795-0.933)	0.917 (0.909-0.924)
	Referable DR	0.927 (0.915-0.939)	0.928 (0.905-0.952)	0.926 (0.919-0.932)
**Datasets with portable fundus images**				
**CPSSDRE**	Referable DR	0.908 (0.905-0.911)	0.905 (0.899-0.911)	0.911 (0.909-0.913)
**CPSSDRM**		0.896 (0.891-0.902)	0.891 (0.880-0.902)	0.901 (0.897-0.905)
**CPSSDRW**		0.906 (0.897-0.913)	0.902 (0.885-0.916)	0.909 (0.905-0.914)
**CPSSDRN**		0.920 (0.892-0.943)	0.939 (0.891-0.980)	0.901 (0.877-0.924)
**ADRS**		0.909 (0.884-0.931)	0.906 (0.860-0.949)	0.912 (0.896-0.926)
**UDRS**		0.899 (0.879-0.918)	0.902 (0.862-0.937)	0.896 (0.885-0.907)

Abbreviations: DR, diabetic retinopathy; AUC, area under the receiver operating characteristic curve; CI, confidence interval; NPDR, non-proliferative diabetic retinopathy; PDR, proliferative diabetic retinopathy; DME, diabetic macular edema; NDSP, Nicheng Diabetes Screening Project; DRPS, Diabetic Retinopathy Progression Study; WTHM, Wuhan Tongji Health Management; PUDM, Peking Union Diabetes Management; CNDCS, China National Diabetic Complications Study; GDES, Guangzhou Diabetic Eye Study; CUHK-STDR, Chinese University of Hong Kong-Sight-Threatening Diabetic Retinopathy; SEED, Singapore Epidemiology of Eye Diseases; SiDRP, Singapore National Diabetic Retinopathy Screening Program; SN-DREAMS, Sankara Nethralaya-Diabetic Retinopathy Epidemiology and Molecular Genetics Study; TNDRSP, Thai National Diabetic Retinopathy Screening Program; UKB, United Kingdom Biobank; CPSSDRE, Chinese Portable Screening Study for Diabetic Retinopathy-East; CPSSDRM, Chinese Portable Screening Study for Diabetic Retinopathy-Middle; CPSSDRW, Chinese Portable Screening Study for Diabetic Retinopathy-West; CPSSDRN, Chinese Portable Screening Study for Diabetic Retinopathy-Northeast; ADRS, Algerian Diabetic Retinopathy Study; UDRS, Uzbek Diabetic Retinopathy Study.

These evaluations were performed using the samples reported in Table S1 (standard fundus images) and Table S2 (portable fundus images).

Supplementary Table 8. Example of management recommendations to one case in the retrospective evaluation of the LLM module.

	Example case, model answer and four responses	Evaluation Domains		
		**Extent of inappropriate content**	**Extent of missing content**	**Likelihood of possible harm**
**Case**	Age: 57.<br>Sex: Female.<br>Smoking status: Occasional.<br>Drinking status: Drinking alcohol when socializing.<br>Eating habits: Balanced meat and vegetables.<br>Physical activity: Running every day for 60 minutes.<br>History of the endocrine system: Elevated fasting plasma glucose was detected in 2005, and then she was diagnosed with type 2 diabetes. Now she is injecting insulin (15U Lantus at night), and taking repaglinide orally, with a slightly controlled diet. In 2000, she was diagnosed with dyslipidemia (Triglyceride: 4.63 mmol/L in 2013, 6.75 mmol/L in 2015, 4.92 mmol/L in 2016; Cholesterol: 6 mmol/L in 2013, 6.28 mmol/L in 2015, 6.27 mmol/L in 2016; Low-density lipoprotein cholesterol: 3.58mmol/L in 2016). She is now taking fenofibrate and atorvastatin intermittently, with a slightly controlled diet.<br>History of elevated blood pressure: In 2005, elevated blood pressure was detected, which once reached 180/110mmHg. Now taking valsartan for treatment, the usual blood pressure is around 130/85mmHg. She denies a history of stroke.<br>History of the circulatory system: In 2014, color Doppler ultrasound showed rough carotid intima and thickened carotid intima-media layer.<br>Family history: Her father has hypertension. One of her brothers has diabetes, and the other has a history of rectal cancer and kidney cancer.<br>Systolic blood pressure: 130 mmHg.<br>Diastolic blood pressure: 78 mmHg.<br>Heart rate: 72 beats per minute.<br>Serum triglycerides: 3.21 mmol/L.<br>Total cholesterol: 4.95 mmol/L.	-	-	-

	High-density lipoprotein cholesterol: 1.14 mmol/L.<br>Low-density lipoprotein cholesterol: 2.72 mmol/L.<br>Urine albumin-to-creatinine ratio: 33.41 mg/g.<br>Estimated glomerular filtration rate: 41.44 mL/min/1.73m^2.<br>Fasting plasma glucose: 8.94 mmol/L.<br>Glycated hemoglobin: 10.5%.<br>Aspartate aminotransferase: 19.7 U/L.<br>Alanine aminotransferase: 16.5 U/L.<br>Gamma-glutamyl transferase: 21 U/L.<br>DR grade: 0 (DR not present).<br>DME grade: 0 (DME not present).			
**Model answer**	1. Blood sugar control: The patient's fasting blood sugar and glycosylated hemoglobin indicators are high, indicating poor blood sugar control. Under the guidance of an endocrinologist, adjust the dose or type of insulin, adjust the dose or type of oral hypoglycemic drugs, and adjust the diet as needed. If possible, it is recommended to communicate with a diabetes specialist as soon as possible to manage blood sugar more effectively.<br><br>2. Blood pressure control: Although the patient has a history of hypertension, the current blood pressure is basically within the normal range, and should continue to take valsartan to treat hypertension. Also pay attention to limiting salt intake to control BP < 130/80 mmHg.<br><br>3. Blood lipid control: The patient's triglycerides, total cholesterol, and low-density lipoprotein indicators are high, and more active blood lipid control is required. You can consider adjusting the drug regimen of fenofibrate and atorvastatin, and at the same time, pay attention to diet control and appropriately limit the intake of fat and sugar.<br><br>4. Diet management: patients should maintain a healthy diet, control carbohydrate and fat intake, and eat more vegetables, whole grains and lean meat.	-	-	-

	5. Exercise management: The patient has a good exercise habit of walking for 60 minutes a day. It is recommended to maintain and appropriately increase the time and intensity of exercise.<br><br>6. Ophthalmic examination: The patient has no obvious DR and DME at present. It is recommended to have a yearly follow-up for fundus examination. If there is any ocular symptom (such as visual symptoms), follow-up with an ophthalmologist is advised.<br><br>7. Cardiovascular disease reduction and management: Patients with a history of diabetes, hypertension, and dyslipidemia should prevent the occurrence of cardiovascular disease. Consider GLP1-RA or SGLT2i to achieve better cardiorenal outcomes.<br><br>8. Renal protection: According to the eGFR/ACR value, it is recommended to continue to regularly monitor renal function and treat. The UACR is > 30, thus there should be a consideration for a repeat of this test in three months' time. If this is still >30, an ACEI/ARB and possibly a SGLT2i should be considered.<br><br>9. Consider quitting smoking to prevent progression of diabetes-related complications.			
**DeepDR-LLM (the LLM module)**	To improve blood sugar control, the following are recommended comprehensive diabetes management measures:<br><br>1. Dietary control: limit the intake of high-sugar, high-fat, high-salt, and high-calorie foods, and increase the intake of vegetables, fruits, whole grains, and protein sources (such as meat) and soy products) and avoid excessive intake of beverages high in saturated fat, carbohydrates, and sugar. Develop a personalized meal plan based on personal physical condition, gender, age and other factors, and pay attention to the intake of sufficient fiber, dietary fiber and low GI foods.<br><br>2. Moderate exercise: Insist on at least 150 minutes of moderate-intensity aerobic exercise per week.	None	None	Low

3. Blood sugar, blood pressure and blood lipid control: The patient's fasting blood sugar and glycosylated hemoglobin indicators are relatively high, indicating poor blood sugar control. The islet function test should be completed under the guidance of an endocrinologist, the insulin dose or dosage form should be adjusted, and oral hypoglycemic medications should be adjusted. If possible, it is recommended to communicate with a diabetes specialist as soon as possible to manage blood sugar more effectively. Considering adding GLP1-RA or SGLT2i to achieve better glycemic control. Although the patient has a history of hypertension, his current blood pressure is basically within the normal range, and he should continue to take valsartan for the treatment of hypertension. The patient's triglycerides, total cholesterol, and low-density lipoprotein indicators are higher, and more active blood lipid control is needed. Consider adjusting the dose of fenofibrate and atorvastatin to achieve better blood lipid control.

4. Control the total energy intake: reasonably distribute the daily energy intake.

5. Regularly monitor indicators: Regularly test indicators such as blood sugar, blood pressure, and blood lipids, and adjust them according to the diabetes treatment plan guided by the doctor.

6. Actively participate in treatment: Cooperate with drug treatment, adjust eating habits, change bad living habits (consider quitting smoking to prevent the progression of diabetes related complications and quitting drinking to prevent alcohol toxicity), and follow treatment instructions. This is the key to successful treatment of diabetes.

7. Psychological stress management: maintain a happy state and mood, and avoid excessive anxiety and depression.

	8. Regular physical examination: According to personal risk and doctor's advice, conduct routine examinations such as fundus examination, blood routine screening, liver function test, and renal function test.<br><br>9. Screening for diabetic nephropathy: The eGFR value of the patient decreases moderately, indicating that there may be kidney damage caused by diabetes. Regular monitoring of renal function is recommended.<br><br>10. Diabetic retinopathy screening: The patient currently has no obvious diabetic retinopathy and diabetic macular edema. It is recommended to follow up the fundus once a year, and follow up with ophthalmologists when ocular discomfort (such as visual symptoms) occurs.			
**LLaMA**	As a medical specialist, I suggest this patient be given medication such as metformin, acarbose and pioglitazone along with regular physical exercises like jogging, cycling etc., so that his condition can improve gradually over time without any serious complications developing.	None	Present, substantial clinical significance	Medium
**PCP**	1. Control of blood sugar: Fasting sugar and HbA1c still not up to control, maybe stopping repaglinide and add SGLT2i for renal protection and glucose control. Optimize the basal insulin - Lantus.<br><br>2. Control of blood pressure: maintain BP below 130/80 mmHg.<br><br>3. Control of blood lipids: aim LDL to 1.8 therefore need to maximize the statin and diet control.<br><br>4. Diet and exercise: maintain healthy diet and exercise, ensure 60 min of moderate intensity diet.<br><br>5. Smoking: advice to stop smoking, assess stage of change in view of smoking.<br><br>6. Diabetes retinopathy screening: ensure yearly screening.	None	Present, substantial clinical significance	Medium

**Resident**	You have 3 major issues, namely sugar control, cholesterol and reducing your chance of having heart disease.<br><br>For diabetes, you need to make sure you are compliant to your prescribed medications. Please tell me if you are having issues with insulin injection technique, sugar monitoring or hypoglycemia. I will refer you to a diabetic nurse to assist you with these issues, including having a hypoglycemia action plan.<br><br>At the same time, you will require more insulin as your HbA1c is high. We would like to aim for a HbA1c of less than 7%, which will reduce your risk of complications such as heart disease, kidney disease and stroke. The fastest way to reduce your HbA1c is with intensification of insulin therapy. I would like to start you on premeal short acting insulin, at a dose of 0.1u/kg/meal. Please take this before meals. I would also like to increase your dose of Lantus to 18u as your fasting sugar readings are still high.<br><br>While you monitor your own sugar at home, we will target a capillary sugar of less than 7mmol/l before meals, and <10mmol/L 2 hours after meals.<br><br>During your next follow up, you will benefit from addition of another medication called empagliflozin which will reduce your risk of developing worsening kidney disease, as I see you already have kidney impairment and protein in your urine.<br><br>With regards to your cholesterol, you will need to take your cholesterol medication daily. If there are any issues with the medications, for example if you are experiencing discomfort when on 2 agents, please let me know. I will review your doses, and if your LDL and triglycerides are still high despite daily doses of atorvastatin and fenofibrate, I would consider changing or adding a different medication. We can review this during your next follow up.<br><br>We will need to address your weight and quit smoking as well, as diabetes management cannot be limited to medications alone. I am glad you are having regular exercise. Running is considered	None	Present, little clinical significance	Low

a moderate to intense activity, depending on your speed. I encourage you to have at least 150 minutes per week of this, although you can slowly work your way up to it. This will assist in weight loss.

You will need to be seen by a dietician as we should plan to have you on a calorie deficit diet to facilitate weight loss. This will not only improve your glucose control, but also reduce the risk of you having heart attacks.

I am pleased that you have managed to get your blood pressure under control. Keep up the good work and continue to be compliant to your medications.

We have covered a lot today. Diabetes is a journey. Do not feel disheartened at high sugar readings. With compliance to medications and lifestyle modifications, diabetes can be well controlled. Please do come back for your follow up appointments so we can discuss your condition further.

Abbreviations: BP, blood pressure; DR, diabetic retinopathy; DME, diabetic macular edema; GLP1-RA, glucagon-like peptide-1 receptor agonists; SGLT2i, sodium-glucose cotransporter 2 inhibitor; eGFR, estimated glomerular filtration rate; UACR, urine albumin-to-creatinine ratio; ACEI, angiotensin converting enzyme inhibitors; ARB, angiotensin receptor blocker; HbA1c, glycated hemoglobin; LDL, low-density lipoprotein; PCP, primary care physician.

Supplementary Table 9. Adjudication process and inter-rater reliability of DR and DME grading.

Dataset	Adjudication process	Inter-rater reliability
**Datasets with standard fundus images**		
**Internal dataset**	Each retinal photograph was graded by two authorized ophthalmologists. The third ophthalmologist who served as the senior supervisor confirmed or corrected when the diagnostic results were contradictory.	Cohen's Kappa: 0.81
**NDSP**		Cohen's Kappa: 0.87
**DRPS**		Cohen's Kappa: 0.83
**WTHM**		Cohen's Kappa: 0.91
**PUDM**		Cohen's Kappa: 0.90
**CNDCS**		Cohen's Kappa: 0.84
**GDES**		Cohen's Kappa: 0.86
**CUHK-STDR**	All anonymized paired retinal photographs were labeled by two well-trained graders, with more than three-year grading experience respectively and an inter-rater reliability over 90%, on full-screen, high-resolution 27-inch monitors (Koninklijke Philips N.V.) in the CUHK Reading Centre, following the international clinical diabetic retinopathy and diabetic macular edema disease severity scales. If there were any grading discrepancies between the two graders, a senior grader with grading experience of more than 10 years would make adjudication.	Cohen's Kappa: 0.92
**SEED**	The primary grader will grade all cases submitted into the system and will escalate all abnormal cases to a secondary grader, as well as 10% of cases graded as normal by the primary grader as a quality audit. For cases where the graders disagree, the case is escalated to an arbitrating grader (>10 years of grading experience) who will make the final decision.	Cohen's Kappa: 0.917

**SiDRP**	The primary grader will grade all cases submitted into the system and will escalate all abnormal cases to a secondary grader, as well as 10% of cases graded as normal by the primary grader as a quality audit. For cases where the graders disagree, the case is escalated to an arbitrating grader (a clinician) who will make the final decision.	Cohen's Kappa: 0.917
**SNDREAMS**	The photographs were graded by two independent observers (ophthalmologists) in a masked fashion. If there was any discrepancy in the grading between both graders, the images were shown to another independent observer (ophthalmologist) whose grade was considered final.	Cohen's Kappa: 0.83
**TNDRSP**	First, both retinal specialists in a group independently graded each image. Then, until consensus was reached, the retinal specialists took turns revising their grades, each time with access to their previous grade and the other retinal specialist's grade, as well as any additional comments about the case either retinal specialist left. If there was still disagreement after each grader had graded the image three times in this way, then the image's reference standard was determined independently by a separate, senior retinal specialist. For grading DR severity levels, differences between no DR and mild NPDR were not adjudicated in order to focus adjudication time on referable disease.	Percentage of agreement for referable DR: 88.43% Percentage of agreement for DME: 88.14%
**UKB**	Each retinal photograph was graded by two authorized ophthalmologists. The third ophthalmologist who served as the senior supervisor confirmed or corrected when the diagnostic results were contradictory.	Cohen's Kappa: 0.82
**Datasets with portable fundus images**		
**NDSP**	Each retinal photograph was graded by two authorized ophthalmologists. The third ophthalmologist who served as the senior supervisor confirmed or corrected when the diagnostic results were contradictory.	Cohen's Kappa: 0.91
**CPSSDRE**		Cohen's Kappa: 0.80
**CPSSDRM**		Cohen's Kappa: 0.82

**CPSSDRW**	Cohen's Kappa: 0.85
**CPSSDRN**	Cohen's Kappa: 0.93
**ARDS**	Cohen's Kappa: 0.89
**UDRS**	Cohen's Kappa: 0.84

Abbreviations: DR, diabetic retinopathy; AUC, area under the receiver operating characteristic curve; CI, confidence interval; NPDR, non-proliferative diabetic retinopathy; PDR, proliferative diabetic retinopathy; DME, diabetic macular edema; NDSP, Nicheng Diabetes Screening Project; DRPS, Diabetic Retinopathy Progression Study; WTHM, Wuhan Tongji Health Management; PUDM, Peking Union Diabetes Management; CNDCS, China National Diabetic Complications Study; GDES, Guangzhou Diabetic Eye Study; CUHK-STDR, Chinese University of Hong Kong-Sight-Threatening Diabetic Retinopathy; SEED, Singapore Epidemiology of Eye Diseases; SiDRP, Singapore National Diabetic Retinopathy Screening Program; SN-DREAMS, Sankara Nethralaya-Diabetic Retinopathy Epidemiology and Molecular Genetics Study; TNDRSP, Thai National Diabetic Retinopathy Screening Program; UKB, United Kingdom Biobank; CPSSDRE, Chinese Portable Screening Study for Diabetic Retinopathy-East; CPSSDRM, Chinese Portable Screening Study for Diabetic Retinopathy-Middle; CPSSDRW, Chinese Portable Screening Study for Diabetic Retinopathy-West; CPSSDRN, Chinese Portable Screening Study for Diabetic Retinopathy-Northeast; ADRS, Algerian Diabetic Retinopathy Study; UDRS, Uzbek Diabetic Retinopathy Study.

Supplementary Table 10. Characteristics of the paired clinical data and real-world health management recommendations included in the dataset for supervised fine-tuning of the LLM module.

Characteristics	Dataset for supervised fine-tuning†
Age (year)	47.16 ± 11.83
Male, n (%)	235,772 (63.42%)
Diabetes, n (%)	40,173 (10.81%)
Hypertension, n (%)	109,897 (29.56%)
Diabetic retinopathy, n (%)*	5,166 (12.86%)
Diabetic macular edema, n (%)*	308 (0.77%)
Hypertensive retinopathy, n (%)	73,423 (19.75%)
Cataract, n (%)	19,220 (5.17%)
Age-related macular degeneration or AMD suspect, n (%)	5,115 (1.38%)
Character count of input clinical data	410.01 ± 62.67
Character count of output management recommendations	622.20 ± 105.33

Data were presented as “mean ± standard deviations” for continuous variables, or “number of cases (%)” for categorical variables.

The diagnosis of diabetic retinopathy, diabetic macular edema, cataract, and age-related macular edema was extracted from the clinical data.

* The percentage of diabetic retinopathy and diabetic macular edema was reported based on the number of cases with diabetes.

† These statistics were calculated based on 371,763 paired clinical data and real-world management recommendations.

Supplementary Table 11. Summary of laboratory tests and fundus examinations in the real-world prospective study.

Item	Method	Equipment used
**Laboratory tests**		
FPG (mmol/L)	Glucose oxidase or hexokinase method	Roche cobas c702
HbA1c (%)	High-performance liquid chromatography	Premier Hb9210
Lipid profile	Enzymatic method	Roche cobas c702
Kidney function	Enzymatic method	Roche cobas c702
Liver function	Enzymatic method	Roche cobas c702
**Fundus examination**		
Digital fundus images	Two-field (macula-centered and optic disc-centered)<br>45° images<br>No pupillary dilation<br>Original image resolution: 3264 × 2448 pixels	Topcon TRC-NW400

Abbreviations: FPG, fasting plasma glucose; HbA1c, glycated hemoglobin.

Supplementary Table 12. Characteristics of the primary care physicians involved in the real-world prospective study.

Characteristics	PCPs involved
Age (year)	38.5 (33.25, 45.00)
Male, n (%)	2 (16.67%)
Years of practice in primary care (year)	17 (8.75, 24.25)

Data were presented as “median (interquartile range)” for continuous variables or “number of individuals (%)” for categorical variables.

A Real-world Prospective Study on Diabetes Management Assistance System based on Large Language Model

STUDY PROTOCOL

Huadong Sanatorium

Version submitted to Ethics Committee on 29 March 2023

Contents

1. Study Background…………………………………………………………………………3
2. Specific Aims…………………………………………………………………………...…4
3. Study Design………………………………………………………………………………4
   3.1 Overall study design……………………………………………………………...…4
      3.1.1 Sub-study I: Patients with newly diagnosed diabetes………………………………5
      3.1.2 Sub-study II: Patients with referable DR…………………………………………5
      3.1.3. Exploratory studies……………………………………………………………...5
   3.2 Sample size estimation………………………………………………………………...6
4. Diagnosis, Inclusion, and Exclusion Criteria……………………………………………6
   4.1 Diagnosis criteria……………………………………………………………………...6
   4.2 Inclusion criteria………………………………………………………………………6
   4.3 Exclusion criteria……………………………………………………………………...6
5. Recruitment……………………………………………………………………………….6
6. Study Outcomes…………………………………………………………………………...7
   6.1 Sub-study I……………………………………………………………………………7
      6.1.1 Primary outcome…………………………………………………………………7
      6.1.2 Secondary outcomes……………………………………………………………..7
   6.2 Sub-study II…………………………………………………………………………...7
      6.2.1 Primary outcome…………………………………………………………………7
      6.2.2 Secondary outcome………………………………………………………………7
   6.3 Exploratory outcomes…………………………………………………………………7
      6.3.1 Post-deployment evaluation of management recommendations in the PCP+DeepDR-LLM arm……………………………………………………………………………7
      6.3.2 Satisfaction questionnaire for PCPs (using the Likert scale for the following seven items) …………………………………………..……………………………...…7
7. Data collection…………………………………………………………………………….8
   7.1 Baseline data collection……………………………………………………………….8
   7.2 Sub-study I…………………………………………………………………………….8
   7.3 Sub-study II…………………………………………………………………………...8
   7.4 Exploratory studies……………………………………………………………………8
      7.4.1 Post-deployment evaluation of management recommendations in the PCP+DeepDR-LLM arm…………………………………………………….…………………8
      7.4.2 Satisfaction questionnaire for PCPs (using the Likert scale) …………………………8
8. Statistical Analysis………………………………………………………………………...9
   8.1 Sub-study I…………………………………………………………………………….9
   8.2 Sub-study II…………………………………………………………………………...9
   8.3 Exploratory studies……………………………………………………………………9
9. References…………………………………………………………………………………9

1. Study Background

The increasing prevalence of diabetes, its complications, and related substantial economic burden make diabetes a significant health challenge, particularly in low-resource settings[1-4]. In 2021, 536.6 million people had diabetes worldwide, with 80.6% from low-income and middle-income countries (LMICs)[2]. In these countries, insufficient healthcare infrastructure (such as screening tools for diabetic complications) and a lack of trained primary care physicians (PCPs) are the principal barriers faced by underserved populations for primary diabetes care, resulting in many individuals with diabetes under-diagnosed, inappropriately referred, or poorly managed[3].

Of all the diabetes complications, diabetic retinopathy (DR) is the most common, and represents still the leading cause of blindness in working-aged adults worldwide. DR affects 30 to 40% of individuals with diabetes[5-7], and thus, globally, more than 100 million individuals have DR[5,8]. The presence of DR also signifies a heightened risk of other complications (e.g., kidney, cardiovascular)[9]. Thus, DR screening is an indispensable element of diabetes care. However, in low-resource settings, where DR screening programs are nonexistent or sparse, and appropriate and timely access to specialized ophthalmic care for those needing referral is limited, the visual burden of DR is particularly high[10,11], extending beyond individual health with socioeconomic implications[12]. A key strategy is to have a sustainable DR screening program coupled with sufficiently trained PCPs to manage such a program, understand the disease, and make appropriate referrals and recommendations for different states of diabetes and severity of DR based on international guidelines[13]. Several confluent technologies have enabled this unmet need to be increasingly met, including telemedicine development with wireless mobile infrastructure[14-16], artificial intelligence (AI) deep learning (DL) models based on retinal images[17-19], and the development of low-cost and handheld portable mobile devices[20,21]. However, despite these major advances, a key limitation remains the lack of sufficiently trained PCPs who could manage and undertake evidence-based DR screening tasks in low-resource settings[22].

Prior AI models in diabetes care management offered several advantages, such as data analysis, decision support and even some brief patient education but faced limitations in natural language understanding and personalized treatment[23]. Large language models (LLMs) have now shown substantial promise in increasing various aspects of healthcare, offering a more comprehensive understanding of the medical literature, patient data, and individualized care requirements[24]. LLMs have thus the potential to optimize patient monitoring, personalization of treatment plans, and patient education, leading to potentially improved outcomes for patients with diabetes. Although current studies have already shown the promising potential of LLMs in generating answers to real-world consumer queries for medical information, these LLMs cannot provide reliable and detailed management recommendations for patients with specific diseases, including diabetes[24,25].

We and others have previously developed several image-based DL systems for DR screening based on fundus images taken from standard retinal cameras[17-19]. To address critical gaps in real-world diabetes care in under-resourced settings, we developed a novel integrated system combining DL with LLM (DeepDR-LLM) to provide “end-to-end” comprehensive primary diabetes care and DR

screening with patient management recommendations for untrained PCPs to manage patients with diabetes.

To address these gaps, we introduce an innovative digital solution - DeepDR-LLM - a system that integrates a LLM module with an image-based DL module to offer a comprehensive approach for primary diabetes care and DR screening. Our system is tailored for PCPs, particularly those working in high-volume and low-resource settings. Essentially, the DeepDR-LLM system comprises two core components: a LLM module and an image-based deep learning module, referred to as DeepDR-Transformer. To validate the effectiveness of our DeepDR-LLM system, we propose to conduct a two-arm, real-world, four-week prospective study in a high-volume primary care setting, deploying the integrated DeepDR-LLM system. We will compare diabetes management adherence between patients under the care of unassisted PCPs and those under the care of PCPs assisted by DeepDR-LLM.

2. Specific Aims

We aim to conduct a two-arm, real-world, four-week prospective study in a high-volume primary care setting, deploying the integrated DeepDR-LLM system. In the first arm (named unassisted PCP arm), PCPs give management recommendations to patients with diabetes without the assistance of DeepDR-LLM; while in the second arm (PCP+DeepDR-LLM arm), PCPs give management recommendations to patients with diabetes with the assistance of DeepDR-LLM. We aim to conduct two sub-studies in this real-world study: 1) Sub-study I: patients with newly diagnosed diabetes; 2) Sub-study II: patients diagnosed with referable DR. In two sub-studies, we will compare the patient outcomes between two arms. Moreover, we will conduct several exploratory studies. In the PCP+DeepDR-LLM arm, we will conduct comparison evaluations on the management recommendations given by unassisted PCPs, DeepDR-LLM, and PCP+DeepDR-LLM. Additionally, we will ask PCPs who participated in the PCP+DeepDR-LLM arm of the real-world prospective study to complete a user satisfaction questionnaire to capture the PCPs' perceptions and satisfactions towards the DeepDR-LLM system.

3. Study Design

3.1 Overall study design

Participants attending the health examinations in Huadong Sanatorium will receive medical history taking, physical examinations, laboratory tests, and fundus examinations. In this study, a total of 12 PCPs will be responsible for primary diabetes care management. Participants will be divided into two arms (the unassisted PCP arm and the PCP+DeepDR-LLM arm) according to the visit time of the participant. Participants attending health examinations during the first 6 weeks of the evaluation period will be included in the unassisted PCP arm, while those during the later 6 weeks of the evaluation period) will be included in the PCP+DeepDR-LLM arm. In the unassisted PCP arm, based on examination results, PCPs will give management recommendations. In the PCP+DeepDR-LLM arm, the DeepDR-LLM system will be integrated into the clinical workflow.

Initially, PCPs give management recommendations independently. Then, the DeepDR-LLM system assists PCPs in generating DR/DME diagnosis results, and utilizes DR/DME diagnosis results and patient information from the electronic health systems, including medical history, physical examinations, and laboratory tests to automatically generate recommendations. Subsequently, PCPs edit and produce their final recommendations by taking DeepDR-LLM's recommendations into account. In both arms, participants will be given treatment advice for diabetes face-to-face by PCPs based on the above recommendations.

These participants will register on the mobile follow-up platform deployed in the study site, which could reach the participants via instant messaging and collect information on their current condition of diabetes management using online questionnaires. They will be followed up through the mobile follow-up platform.

For the training process of PCPs in the real-world prospective study, we will not train them how to provide diabetes management recommendations in both arms. In the first six weeks, PCPs will be told to keep their usual management approaches and routine. Before the integration of the DeepDR-LLM system into clinical workflows of primary diabetes care in the PCP+DeepDR-LLM arm, PCPs will be trained how to use an assistive tool (called "Diabetes Management Assistance System") to help them generate diabetes management recommendations. We will not disclose the name (i.e., DeepDR-LLM), mechanism, and performance of this assistive tool to PCPs. And they will be told that they can accept, reject, or edit the management recommendations generated by this assistive tool, based on their own judgment. After the integration of the DeepDR-LLM system into the clinical workflows, newly generated data will not be used to re-train the DeepDR-LLM system.

3.1.1 Sub-study I: Patients with newly diagnosed diabetes

For all participants with newly diagnosed diabetes in two arms, they will fill out a questionnaire investigating their status of diabetes management at baseline, 2-week follow-up, and 4-week follow-up. The questionnaire investigates the frequency of blood glucose monitoring, physical therapy, nutrient therapy, drug therapy, and cessation of drinking and smoking.

3.1.2 Sub-study II: Patients with referable DR

For all participants diagnosed as referable DR in two arms, they will be contacted at the 2-week follow-up to check whether and when they attend appointments with an ophthalmologist.

3.1.3. Exploratory studies

We will conduct the post-deployment evaluation (ranking, quality, and empathy) of management recommendations in Sub-study I and II of the PCP+DeepDR-LLM arm, which will be provided by three consultant-level endocrinologists and participants. For participants, their opinions on three recommendations will be collected at the 4-week follow-up. Each of the three consultant-level endocrinologists will be invited to evaluate all the cases. For each case, the PCP, DeepDR-LLM, and PCP+DeepDR-LLM's recommendations will be anonymized and randomly ordered. The endocrinologists and surveyed participants, rank these three recommendations and judge both "the quality of information provided" (very poor, poor, acceptable, good, or very good) and "the

empathy or bedside manner provided" (not empathetic, slightly empathetic, moderately empathetic, empathetic, and very empathetic).
Furthermore, PCPs who use the DeepDR-LLM system in this real-world study will be invited to complete a satisfaction questionnaire within two weeks after the conclusion of the study. The questionnaire includes 7 item questions assessing these PCPs' views regarding the integration of DeepDR-LLM into daily routine practice.

3.2 Sample size estimation

Approximately at least 1600 patients with diabetes will be enrolled in this study. This sample size is not based on statistical considerations but will be selected in an effort to include all subjects with diabetes attending the health examination program in the study site in 12 weeks. It is anticipated that each arm (either unassisted PCP arm or PCP+DeepDR-LLM arm) will have a minimum sample size of approximately 800 patients with diabetes.

4. Diagnosis, Inclusion, and Exclusion Criteria

4.1 Diagnosis criteria

DR severity is graded into five levels (non-DR, mild non-proliferative DR [NPDR], moderate NPDR, severe NPDR, or proliferative DR [PDR], respectively), according to the International Clinical Diabetic Retinopathy Disease Severity Scale (AAO, October 2002)[26]. Diabetic macular edema (DME) is considered to be present when there is retinal thickening at or within one disc diameter of the macular center or definite hard exudates in this region[27]. Referable DR is defined as moderate NPDR or worse, DME, or both. Retinal photographs are flagged as ungradable according to our previous study[19]. Diabetes is diagnosed according to the latest ADA guidelines[28].

4.2 Inclusion criteria

- Ethnic Chinese
- Aged 18-70 years old
- With diabetes
- Receiving the digital fundus examinations

4.3 Exclusion criteria

- Participants without gradable fundus images
- Pregnant, breastfeeding women, or women planning to get pregnant
- Participants expected to have poor compliance
- Participants with mental disorders who cannot cooperate

5. Recruitment

Participants attending the health examinations in Huadong Sanatorium during the 12-week evaluation period will receive medical history taking, physical examinations, laboratory tests, and

fundus examinations. Among them, patients with known diabetes and newly diagnosed diabetes will be subsequently recruited and included in this study according to the above inclusion and exclusion criteria. Informed consent will be obtained from these participants.

6. Study Outcomes

6.1 Sub-study I

6.1.1 Primary outcome

- Adherence to anti-diabetic drugs

6.1.2 Secondary outcomes

(1) Changes in eating habits, including refined grains, whole grains, starchy vegetables, beans, fresh vegetables, fresh fruits, etc.

(2) Changes in the consumption of cigarettes

(3) Changes in the consumption of alcohol

(4) Changes in the physical activity

(5) Frequency of blood glucose monitoring

6.2 Sub-study II

6.2.1 Primary outcome

- Whether participants attend appointments with an ophthalmologist

6.2.2 Secondary outcome

When participants attend appointments with an ophthalmologist (among those attending appointments)

6.3 Exploratory outcomes

6.3.1 Post-deployment evaluation of management recommendations in the PCP+DeepDR-LLM arm

(1) Quality of information provided (very poor, poor, acceptable, good, or very good)

(2) Empathy or bedside manner provided (not empathetic, slightly empathetic, moderately empathetic, empathetic, or very empathetic)

6.3.2 Satisfaction questionnaire for PCPs (using the Likert scale for the following seven items: 1->5 for very dissatisfied/disagree -> very satisfied/agree)

(1) I think the DeepDR-LLM system is user-friendly.

(2) I believe the integration of the DeepDR-LLM system into primary diabetes care can help me provide better management recommendations.

(3) I believe the DeepDR-LLM system is safe in both DR grading and management recommendations.

(4) I think the integration of the DeepDR-LLM system into future clinical practice can save my time.

(5) I believe most primary care physicians can learn to use the DeepDR-LLM system quickly.

(6) I would like to use the DeepDR-LLM system in my future practice for primary diabetes care.
(7) Overall, I am satisfied with the DeepDR-LLM system.

7. Data collection
7.1 Baseline data collection

- Medical history: age, sex, smoking status, drinking status, eating habits, physical activity, history of the endocrine system, history of the cardiovascular system, history of elevated blood pressure, family history.
- Physical examinations: height, weight, blood pressure, and heart rate.
- Laboratory tests and fundus examinations

Item	Method	Equipment used
**Laboratory tests**		
FPG (mmol/L)	Glucose oxidase or hexokinase method	Roche cobas c702
HbA1c (%)	High-performance liquid chromatography	Premier Hb9210
Lipid profile	Enzymatic method	Roche cobas c702
Kidney function	Enzymatic method	Roche cobas c702
Liver function	Enzymatic method	Roche cobas c702
**Fundus examination**		
Digital fundus images	Two-field (macula-centered and optic disc-centered)<br>45° images<br>No pupillary dilation<br>Original image resolution: 3264 × 2448 pixels	Topcon TRC-NW400

7.2 Sub-study I

- Baseline questionnaire (attached below)
- 2-week and 4-week follow-up questionnaire (attached below)

7.3 Sub-study II

- Whether and when participants attend appointments with an ophthalmologist at the 2-week follow-up

7.4 Exploratory studies
7.4.1 Post-deployment evaluation of management recommendations in the PCP+DeepDR-LLM arm
(1) By participants at the 4-week follow-up.
(2) By three endocrinologists within two weeks after the study closure.
7.4.2 Satisfaction questionnaire for PCPs (using the Likert scale)
Within two weeks after the study closure.

8. Statistical Analysis

8.1 Sub-study I

To compare the differences in primary and secondary outcomes at 2-week and 4-week follow-up among participants with newly diagnosed diabetes between two arms, we will perform linear mixed models, logistic regression models, and linear regression models.

8.2 Sub-study II

To compare the differences in primary and secondary outcomes at 2-week follow-up among participants with referable DR between two arms, we will perform logistic regression models for the primary outcome and linear regression models for the secondary outcome.

8.3 Exploratory studies

For post-deployment evaluation of management recommendations by both endocrinologists and participants, we will report the percentage of evaluators for their first-choice preference as well as the Clopper-Pearson 95% confidence interval.

9. References

1. Jia W. Diabetes care in China: Innovations and implications. *J Diabetes Investig*. Nov 2022;13(11):1795-1797. doi:10.1111/jdi.13908
2. Sun H, Saeedi P, Karuranga S, et al. IDF Diabetes Atlas: Global, regional and country-level diabetes prevalence estimates for 2021 and projections for 2045. *Diabetes Research and Clinical Practice*. 2022/01/01/ 2022;183:109119. doi:https://doi.org/10.1016/j.diabres.2021.109119
3. Chan JCN, Lim L-L, Wareham NJ, et al. The Lancet Commission on diabetes: using data to transform diabetes care and patient lives. *Lancet*. 2021;396(10267):2019-2082. doi:10.1016/S0140-6736(20)32374-6
4. Bee YM, Tai ES, Wong TY. Singapore's "War on Diabetes". *Lancet Diabetes Endocrinol*. 2022;10(6):391-392. doi:10.1016/S2213-8587(22)00133-4
5. Yau JWY, Rogers SL, Kawasaki R, et al. Global prevalence and major risk factors of diabetic retinopathy. *Diabetes Care*. 2012;35(3):556-564. doi:10.2337/dc11-1909
6. Ruta LM, Magliano DJ, Lemesurier R, Taylor HR, Zimmet PZ, Shaw JE. Prevalence of diabetic retinopathy in Type 2 diabetes in developing and developed countries. *Diabet Med*. 2013;30(4):387-398. doi:10.1111/dme.12119
7. Cheung N, Mitchell P, Wong TY. Diabetic retinopathy. *Lancet*. 2010;376(9735):124-136. doi:10.1016/S0140-6736(09)62124-3
8. Ting DSW, Cheung GCM, Wong TY. Diabetic retinopathy: global prevalence, major risk factors, screening practices and public health challenges: a review. *Clin Exp Ophthalmol*. 2016;44(4):260-277. doi:10.1111/ceo.12696
9. Cheung N, Wong TY. Diabetic retinopathy and systemic vascular complications. *Prog Retin Eye Res*. 2008;27(2):161-176. doi:10.1016/j.preteyeres.2007.12.001
10. Yim D, Chandra S, Sondh R, Thottarath S, Sivaprasad S. Barriers in establishing systematic diabetic retinopathy screening through telemedicine in low- and middle-income countries. *Indian J Ophthalmol*. 2021;69(11):2987-2992. doi:10.4103/ijo.IJO_1411_21

11. Wong TY, Sabanayagam C. Strategies to Tackle the Global Burden of Diabetic Retinopathy: From Epidemiology to Artificial Intelligence. *Ophthalmologica*. 2020;243(1)doi:10.1159/000502387
12. Fenwick E, Rees G, Pesudovs K, et al. Social and emotional impact of diabetic retinopathy: a review. *Clin Exp Ophthalmol*. 2012;40(1):27-38. doi:10.1111/j.1442-9071.2011.02599.x
13. Wong TY, Sun J, Kawasaki R, et al. Guidelines on Diabetic Eye Care: The International Council of Ophthalmology Recommendations for Screening, Follow-up, Referral, and Treatment Based on Resource Settings. *Ophthalmology*. 2018;125(10):1608-1622. doi:10.1016/j.ophtha.2018.04.007
14. Vujosevic S, Aldington SJ, Silva P, et al. Screening for diabetic retinopathy: new perspectives and challenges. *Lancet Diabetes Endocrinol*. 2020;8(4):337-347. doi:10.1016/S2213-8587(19)30411-5
15. Ting DSW, Peng L, Varadarajan AV, et al. Deep learning in ophthalmology: The technical and clinical considerations. *Prog Retin Eye Res*. 2019;72:100759. doi:10.1016/j.preteyeres.2019.04.003
16. Gunasekeran DV, Ting DSW, Tan GSW, Wong TY. Artificial intelligence for diabetic retinopathy screening, prediction and management. *Curr Opin Ophthalmol*. 2020;31(5):357-365. doi:10.1097/ICU.0000000000000693
17. Ting DSW, Cheung CY-L, Lim G, et al. Development and Validation of a Deep Learning System for Diabetic Retinopathy and Related Eye Diseases Using Retinal Images From Multiethnic Populations With Diabetes. *JAMA*. 2017;318(22):2211-2223. doi:10.1001/jama.2017.18152
18. Gulshan V, Peng L, Coram M, et al. Development and Validation of a Deep Learning Algorithm for Detection of Diabetic Retinopathy in Retinal Fundus Photographs. *JAMA*. 2016;316(22):2402-2410. doi:10.1001/jama.2016.17216
19. Dai L, Wu L, Li H, et al. A deep learning system for detecting diabetic retinopathy across the disease spectrum. *Nat Commun*. 2021;12(1):3242. doi:10.1038/s41467-021-23458-5
20. Grauslund J. Diabetic retinopathy screening in the emerging era of artificial intelligence. *Diabetologia*. 2022;65(9):1415-1423. doi:10.1007/s00125-022-05727-0
21. Sheikh A, Bhatti A, Adeyemi O, Raja M, Sheikh I. The Utility of Smartphone-Based Artificial Intelligence Approaches for Diabetic Retinopathy: A Literature Review and Meta-Analysis. *J Curr Ophthalmol*. 2021;33(3):219-226. doi:10.4103/2452-2325.329064
22. Burton MJ, Ramke J, Marques AP, et al. The Lancet Global Health Commission on Global Eye Health: vision beyond 2020. *Lancet Glob Health*. Apr 2021;9(4):e489-e551. doi:10.1016/S2214-109X(20)30488-5
23. Contreras I, Vehi J. Artificial Intelligence for Diabetes Management and Decision Support: Literature Review. *J Med Internet Res*. 2018;20(5):e10775. doi:10.2196/10775
24. OpenAI. GPT-4 Technical Report. *ArXiv*. 2023;abs/2303.08774
25. Touvron H, Lavril T, Izacard G, et al. Llama: Open and efficient foundation language models. *arXiv preprint arXiv:230213971*. 2023;
26. Wilkinson C, Ferris FL, Klein RE, et al. Proposed international clinical diabetic retinopathy and diabetic macular edema disease severity scales. *Ophthalmology*. 2003;110(9):1677-1682.

27. Photocoagulation for diabetic macular edema. Early Treatment Diabetic Retinopathy Study report number 1. Early Treatment Diabetic Retinopathy Study research group. *Arch Ophthalmol*. 1985;103(12):1796-1806.
28. 2. Classification and Diagnosis of Diabetes: Standards of Medical Care in Diabetes-2022. *Diabetes Care*. 2022;45(Suppl 1):S17-S38. doi:10.2337/dc22-S002
