# Supplementary material for: Integrated image-based deep learning and language models for primary diabetes care
Source: Nat Med. 2024 Jul 19;30(10):2886–96. doi: 10.1038/s41591-024-03139-8 (PMC11485246; doi:10.1038/s41591-024-03139-8)
Supplement: Supplementary file 2 — Reporting Summary [file 41591_2024_3139_MOESM2_ESM.pdf]

Corresponding author(s): Weiping Jia, Yih-Chung Tham, Huating Li, Bin Sheng, and Tien Yin Wong

Last updated by author(s): Jun 14, 2024

Reporting Summary

Nature Portfolio wishes to improve the reproducibility of the work that we publish. This form provides structure for consistency and transparency in reporting. For further information on Nature Portfolio policies, see our Editorial Policies and the Editorial Policy Checklist.

Statistics

For all statistical analyses, confirm that the following items are present in the figure legend, table legend, main text, or Methods section.

n/a	Confirmed	
☐	☒	The exact sample size (*n*) for each experimental group/condition, given as a discrete number and unit of measurement
☐	☒	A statement on whether measurements were taken from distinct samples or whether the same sample was measured repeatedly
☐	☒	The statistical test(s) used AND whether they are one- or two-sided<br>*Only common tests should be described solely by name; describe more complex techniques in the Methods section.*
☒	☐	A description of all covariates tested
☐	☒	A description of any assumptions or corrections, such as tests of normality and adjustment for multiple comparisons
☐	☒	A full description of the statistical parameters including central tendency (e.g. means) or other basic estimates (e.g. regression coefficient) AND variation (e.g. standard deviation) or associated estimates of uncertainty (e.g. confidence intervals)
☐	☒	For null hypothesis testing, the test statistic (e.g. *F*, *t*, *r*) with confidence intervals, effect sizes, degrees of freedom and *P* value noted<br>*Give P values as exact values whenever suitable.*
☒	☐	For Bayesian analysis, information on the choice of priors and Markov chain Monte Carlo settings
☒	☐	For hierarchical and complex designs, identification of the appropriate level for tests and full reporting of outcomes
☒	☐	Estimates of effect sizes (e.g. Cohen's *d*, Pearson's *r*), indicating how they were calculated

Our web collection on statistics for biologists contains articles on many of the points above.

Software and code

Policy information about availability of computer code

Data collection	No special software was used for data collection.
Data analysis	Python version 3.9.18 (Python Software Foundation, Delaware, United States) was used for all statistical analyses in this study. The following third-party python packages were used: Pytorch version 2.0.1 (Facebook, Massachusetts, United States) was used for deep network computing. Scikit-learn version 1.3.2 (David Cournapeau, California, United States) was used for calculating AUC.<br><br>The code being used in the current study for developing the algorithm is provided at https://github.com/DeepPros/DeepDR-LLM.

For manuscripts utilizing custom algorithms or software that are central to the research but not yet described in published literature, software must be made available to editors and reviewers. We strongly encourage code deposition in a community repository (e.g. GitHub). See the Nature Portfolio guidelines for submitting code & software for further information.

Data

Policy information about availability of data

All manuscripts must include a data availability statement. This statement should provide the following information, where applicable:

- Accession codes, unique identifiers, or web links for publicly available datasets
- A description of any restrictions on data availability
- For clinical datasets or third party data, please ensure that the statement adheres to our policy

Individual-level patient data can be accessible with the informed consent of the Data Management Committee from institutions and are not publicly available. Interested investigators can obtain and certify the data transfer agreement and submit requests to Tien Yin Wong (wongtienyin@tsinghua.edu.cn). Investigators who consent to the terms of the data transfer agreement, including, but not limited to, the use of these data only for academic purposes, and to protect the confidentiality of the data and limit the possibility of identification of patients, will be granted access. Requests will be evaluated on a case-by-case basis within one month before receipt of a response. All data shared will be de-identified. For the reproduction of our algorithm code, we have also deposited a minimum dataset at Zenodo (https://zenodo.org/records/11501225), which is publicly available for scientific research and non-commercial use.

Research involving human participants, their data, or biological material

Policy information about studies with human participants or human data. See also policy information about sex, gender (identity/presentation), and sexual orientation and race, ethnicity and racism.

Reporting on sex and gender	Stratified data analysis was reported by sex and the cohort demographics were described in Extended Data Table 4 and Supplementary Tables 1, 2, 3, 10.
Reporting on race, ethnicity, or other socially relevant groupings	For the development and validation of DeepDR-Transformer's performance, a multi-ethnic dataset was used. The reporting on race, ethnicity, or other socially relevant groupings was shown in Supplementary Tables 1-2.<br><br>For other data, the participants were all Chinese, as shown in Extended Data Table 4, Supplementary Table 3, and Supplementary Table 10.
Population characteristics	Detailed cohort characteristics given in Extended Data Table 4, Supplementary Tables 1, 2, 3, 10.
Recruitment	For management recommendations, the language module was fine-tuned on LLaMA using 371,763 real-world management recommendations from 267,730 subjects.<br><br>To investigate the DeepDR-LLM system's ability to give comprehensive management recommendations for patients with diabetes compared with LLaMA and clinicians, we curated a retrospective dataset comprising 100 cases randomly selected from CNDCS.<br><br>For the development and validation of DeepDR-Transformer's performance, a multi-ethnic dataset that comprised a total of 1,085,295 standard fundus images and 161,840 portable fundus images was used.<br><br>In order to further demonstrate the patient outcome of the integration of DeepDR-LLM with clinical and digital workflows, we conducted a real-world prospective study in Huadong Sanatorium. In total, 1,994 participants with diabetes were recruited and included in the study.<br><br>The data for the model training collected from Chinese subjects, might not be representative for the generalized population, potentially introducing biases.
Ethics oversight	This study involves human participants and was approved by the Ethics Committee of Shanghai Sixth People's Hospital (2019-087, approved 29 August 2019; 2023-KY-023(K), approved 7 March 2023; 2023-KY-123(K), approved 5 September 2023) and Huadong Sanitorium (2023-08, approved on 2023-04-02).

Note that full information on the approval of the study protocol must also be provided in the manuscript.

Field-specific reporting

Please select the one below that is the best fit for your research. If you are not sure, read the appropriate sections before making your selection.

☒ Life sciences ☐ Behavioural & social sciences ☐ Ecological, evolutionary & environmental sciences

For a reference copy of the document with all sections, see nature.com/documents/nr-reporting-summary-flat.pdf

Life sciences study design

All studies must disclose on these points even when the disclosure is negative.

Sample size	A specific sample size calculation was not done.<br><br>For management recommendations, the LLM module was fine-tuned on LLaMA using 371,763 real-world management recommendations

from 267,730 subjects.

To investigate the DeepDR-LLM system's ability to give comprehensive management recommendations for patients with diabetes compared with LLaMA and clinicians, we curated a retrospective dataset comprising 100 cases randomly selected from CNDCS.

For the development and validation of DeepDR-Transformer's performance, a multi-ethnic dataset that comprised a total of 1,085,295 standard fundus images and 161,840 portable fundus images was used.

In order to further demonstrate the patient outcome of the integration of DeepDR-LLM with clinical and digital workflows, we conducted a real-world prospective study in Huadong Sanatorium. In total, 1,994 participants with diabetes were recruited and included in the study.

The sample size was determined by the data availability.

Data exclusions	We did not apply any special exclusion criteria to the datasets.
Replication	Replication is not relevant. We used independent validation cohorts to test the models, and the models achieved similar performances in the external validation sets.
Randomization	Samples were randomly allocated to the developmental and testing datasets.
Blinding	During the data processing, all data was first de-identified to remove any patient related information.

Reporting for specific materials, systems and methods

We require information from authors about some types of materials, experimental systems and methods used in many studies. Here, indicate whether each material, system or method listed is relevant to your study. If you are not sure if a list item applies to your research, read the appropriate section before selecting a response.

Materials & experimental systems

n/a	Involved in the study	
☒	☐	Antibodies
☒	☐	Eukaryotic cell lines
☒	☐	Palaeontology and archaeology
☒	☐	Animals and other organisms
☒	☐	Clinical data
☒	☐	Dual use research of concern
☒	☐	Plants

Methods

n/a	Involved in the study	
☒	☐	ChIP-seq
☒	☐	Flow cytometry
☒	☐	MRI-based neuroimaging

Plants

Seed stocks	Not applicable.
Novel plant genotypes	Not applicable.
Authentication	Not applicable.
